# Generative AI in creative education: baseline-linked evidence on learning processes, artistic performance, and authorship

**DOI:** 10.3389/fpsyg.2026.1956939

**Published:** 2026-10-02

**Authors:** Ningtao Shen, Lai Soon Lee, Aneng He

**Affiliations:** 1 School of Animation and Digital Arts, Communication University of Zhejiang, Hangzhou, China; 2 Department of Mathematics and Statistics, Faculty of Science, Universiti Putra Malaysia, Serdang, Malaysia

**Keywords:** art education, artificial intelligence in education, assistive AI, content-generative AI, creative education, development-oriented learning

## Abstract

**Introduction:**

Generative artificial intelligence is increasingly shaping creative learning in higher education art courses, yet its educational value remains contested when creative support, output generation, authorship, and skill development become intertwined. This study investigates AI-supported creative learning in an undergraduate art course.

**Methods:**

The study used linked data from 133 students, including prior course performance, final creative-performance assessment, and post-project questionnaire responses. AI use was operationalized through two dimensions: assistive use for ideation, refinement, process management, and technique learning, and content-generative use involving AI contributions to creative output. The analysis combined baseline-adjusted outcome models, performance-change models, propensity-score adjustment, AI-use intensity comparisons, and robustness checks across alternative AI-use definitions.

**Results:**

The findings show a clear process-outcome decoupling pattern. AI use was associated with stronger perceived support and skill transfer, while final artistic performance and baseline-to-final performance change showed no corresponding improvement. Content-generative AI use was associated with lower evaluated authorship, suggesting that AI-shaped artifacts may reduce the visibility of students' creative decision-making in final outcomes.

**Discussion:**

The study contributes a differentiated account of AI in creative education and argues that responsible AI integration should focus on how students use, document, justify, and reflect on AI within the creative process.

## 1 Introduction

Artificial intelligence (AI) has become part of everyday learning in higher education. Earlier educational technologies had already moved teaching and learning toward more adaptive and data-informed environments (Bond et al., 2019), and AI in education (AIED) has been described as a shift in how learning processes and knowledge construction are organized (Ouyang and Jiao, 2021). The recent spread of generative AI (GenAI) has made this shift more visible. Students can now use AI to search for information, receive feedback, generate ideas, produce images or texts, revise drafts, and complete parts of creative work. These systems therefore enter the space of knowledge production itself, changing how learners create and express ideas (Holmes and Tuomi, 2022). Their educational value remains contested, especially when support for learning becomes closely tied to automation, student autonomy, and the quality of learning engagement (Kasneci et al., 2023).

These questions are especially important in higher education art and design courses. Creative education is not organized only around correct answers or efficient task completion. It also concerns how students develop ideas, make aesthetic judgments, revise their work, and build a sense of authorship. In studio-based learning, originality, personal expression, and creative identity are central educational goals (Winner et al., 2013). In this study, creative learning refers to the process through which students generate, refine, and express original ideas through iterative practice. Creativity involves both originality and effectiveness, which makes human agency and judgment part of the learning outcome rather than a secondary concern (Runco and Jaeger, 2012). Digital tools have long shaped creative learning by changing how students engage with materials and processes (Peppler and Glosson, 2013). GenAI extends this issue because it can participate directly in producing creative content. It may help students explore styles, overcome technical barriers, and move through creative uncertainty, while also making it harder to determine where support ends and substitution begins.

AI use in creative education therefore needs to be examined through its specific forms of participation. Treating AI as a single learning tool hides important differences in how students use it. Different forms of AI interaction can produce different learning experiences (Khosravi et al., 2022). In an art course, AI may function as assistive support when students use it for ideation, reference gathering, process management, refinement, or technique learning. It may function differently when it contributes directly to the content of the final work. The first form can scaffold creative practice; the second can enter the authorship of the work itself. This distinction matters because artistic learning depends on the student's participation in generating, selecting, modifying, and justifying creative decisions.

A central problem is whether AI-supported creative processes lead to stronger educational outcomes. In many educational settings, greater support, smoother workflow, and higher perceived efficiency are often read as signs of better learning. Creative education makes this assumption less secure. Perceived ease may produce an illusion of competence, in which learners feel that they have learned more than their later performance demonstrates (Bjork et al., 2013). Cognitive offloading research also suggests that reliance on external tools can reduce the effort needed for internalization (Risko and Gilbert, 2016). In AI-supported creative learning, students may feel more supported and more productive, while the final work may not show corresponding improvement. Direct AI-generated content may also weaken the perceived authorship of student work. This tension can be understood as a process–outcome decoupling pattern: AI may intensify the visible activity of creative learning without necessarily strengthening the artistic competence, ownership, or judgment through which that learning is evaluated.

This issue also connects to sustainable learning in higher education. Sustainable education concerns more than immediate performance; it emphasizes long-term capability, autonomy, and the capacity to continue learning beyond a single task or course (Rieckmann, 2017). In creative disciplines, this means sustained creative engagement, transferable artistic skills, independent judgment, and confidence in one's own authorship. AI may support these goals when it helps students clarify ideas, learn methods, and reflect on alternatives. It may work against them when students become dependent on generated outputs without developing the ability to explain, adapt, or internalize creative decisions. The key question is how different forms of AI use relate to process support, artistic outcomes, authorship, and longer-term creative development.

This study examines these issues in an undergraduate art course using linked course data from 133 students. The analysis combines students' prior course performance, final creative-performance assessment, and post-project questionnaire responses about AI use and creative learning processes. AI use is examined through two dimensions: assistive AI use and content-generative AI use. The study investigates how these forms of use are associated with multidimensional artistic performance, perceived learning support, creative experience, skill transfer, learner agency, and development-oriented learning outcomes. By linking prior performance with final assessment and process-level questionnaire evidence, the study reduces the limitations of relying on a single post-project survey and provides a more grounded account of how AI enters creative learning.

The study makes three contributions. First, it distinguishes between assistive and content-generative AI use in a domain where that distinction has direct implications for authorship and learning. Second, it connects AI use with both assessed artistic outcomes and students' reported creative-learning processes, allowing the relationship between process experience and performance to be examined rather than assumed. Third, it develops the idea of process–outcome decoupling in AI-supported creative education, showing why AI-enhanced creative activity should be evaluated not only by output quality, but also by authorship, internalization, and transferable creative agency.

## 2 Literature review

The AIED literature provides an important starting point for understanding AI-supported learning, but it also shows why AI use cannot be evaluated only through efficiency, personalization, or system performance. Prior research has examined AI through intelligent tutoring, adaptive learning, automated feedback, learner modeling, learning analytics, and personalized recommendation (Crompton and Burke, 2023; Hwang et al., 2020). These applications have expanded the capacity of educational systems to diagnose learner performance, provide timely guidance, and support data-informed instruction. However, systematic reviews of AI in higher education show that the field has often emphasized system functions and technical implementation more than learners' cognitive, affective, and developmental processes (Bond et al., 2024; Zawacki-Richter et al., 2019). This matters because educational value depends not only on the availability of AI support, but also on how learners interpret, appropriate, and internalize that support. Empirical findings on AI-supported learning outcomes remain mixed, suggesting that AI effects are shaped by task design, learner characteristics, disciplinary context, and assessment methods (Zhai et al., 2021).

The emergence of GenAI extends this issue because AI can now participate in the production of learning artifacts rather than only supporting access, feedback, or adaptation. Empirical studies based on educators' perspectives suggest that GenAI changes how learners interact with tasks, particularly by enabling content generation and task automation (Lee et al., 2024). At the same time, interdisciplinary research highlights both opportunities and risks associated with these technologies, especially in relation to autonomy and learning depth (Kshetri et al., 2023). Recent reviews and meta-analyses provide a similarly balanced picture. ChatGPT-based interventions have been associated with improvements in academic performance, affective-motivational states, and higher-order thinking propensities, but also with reduced reported mental effort (Deng et al., 2025). Broader accounts of GenAI and human learning likewise emphasize that the educational value of GenAI depends on pedagogical framing, assessment design, and learner regulation rather than on tool adoption alone (Yan et al., 2024). These findings suggest that AI should not be treated as a homogeneous educational condition. They also point to the need for evidence that connects students' reported learning experiences with assessed performance rather than relying on perceptions alone.

The tension between support and learning becomes especially visible in research on higher-order thinking and creative problem solving. Student-based studies indicate that GenAI may influence creativity and critical thinking, although such findings are primarily based on self-reported perceptions rather than objective learning outcomes (Alghamdi, 2025). Experimental evidence shows that ChatGPT can improve the quality, elaboration, and originality of students' creative problem-solving outputs, while also making tasks feel easier and less effortful (Urban et al., 2024). This combination is educationally important. Lower effort may help students explore more alternatives, but it may also make it harder for them to judge how much of the performance comes from their own understanding. Recent review evidence suggests that GenAI can support critical and creative thinking when it is embedded in structured and scaffolded learning environments (Li et al., 2026). The emerging literature therefore does not support a simple improvement or harm narrative. Instead, it suggests a process–outcome problem: AI may strengthen the experience of support, fluency, and confidence without necessarily producing equivalent gains in internalized competence.

Studio-based learning research treats creative performance as an iterative and reflective process rather than a single output event. Prior studies emphasize that learning in art and design is embedded in practices of experimentation, critique, revision, originality, authorship, and creative identity (Henriksen, 2016). From a creative cognition perspective, artistic performance is understood as a cycle of idea generation, evaluation, refinement, and expression (Finke et al., 1996; Runco and Jaeger, 2012). GenAI can enter these cycles in several ways. It may support exploration, reference gathering, technical problem solving, and revision, but it may also blur the boundary between process support and content substitution when generated material becomes part of the submitted artifact. Studies of GenAI and creativity show that AI can support idea generation and creative output, especially through recombination and incremental novelty (Lee and Chung, 2024). Research on text-to-image AI and human art production further suggests that generative systems can expand possible outputs while making human filtering, selection, and judgment more important (Zhou and Lee, 2024). In this context, the educational issue is not only output quality, but the student's role in shaping the creative process.

Research in design and creative education has begun to clarify this mechanism. Studies of AI-assisted creative ideation show that GenAI can support idea generation without necessarily reducing all dimensions of creativity, but it may also increase semantic convergence or produce more familiar solutions (Balta-Salvador et al., 2025). Evidence from product design education suggests that AIGC tools can support design ideation, self-efficacy, and learning outcomes when students use them to explore alternatives and refine ideas (Huang et al., 2024). These findings indicate that AI-supported creativity should be understood as a situated learning process rather than a direct tool effect. The same AI system may support creative agency when it is used for exploration, comparison, and revision, but may weaken visible authorship when it substitutes for students' own production decisions.

A key limitation in the existing literature lies in the tendency to treat AI usage as a homogeneous construct. Student-centered studies indicate that different forms of AI usage are associated with different learning experiences (Chan and Hu, 2023). However, many studies still classify students mainly as AI users or non-users, or measure AI use through general frequency, acceptance, or perceived usefulness. This approach hides an important distinction in creative disciplines. AI may function as assistive support when students use it for ideation, reference gathering, process management, refinement, feedback, or technique learning. It may function differently when it contributes directly to visual elements, stylistic features, or other content that appears in the final artifact. The first form can scaffold students' own creative decisions; the second can enter the authorship of the work itself. This distinction is critical because artistic learning depends on students' participation in generating, selecting, modifying, and justifying creative decisions. Despite its importance, empirical research comparing assistive AI use and content-generative AI use remains limited.

Authorship brings this distinction into sharper focus. In art education, authorship is not only a legal or ethical category. It is also an educational indicator of visible creative intention, personal decision-making, and ownership of the work. When AI-generated content becomes part of the final artifact, the student's role may become harder to evaluate from the artifact alone. A student may have actively prompted, selected, edited, recombined, and refined AI outputs, yet those decisions may leave limited visible trace in the final image. Conversely, a polished AI-shaped artifact may conceal limited student transformation or judgment. This creates an assessment problem: evaluators must judge not only visual quality, but also the degree to which the work demonstrates student agency, originality, and process-based learning. Recent discussions of GenAI in higher education assessment similarly argue that AI requires more multifaceted evaluation strategies, including process evidence, reflection, transparency, and alignment between AI use and learning objectives (Yan et al., 2024; Xia et al., 2024).

From a cognitive perspective, AI-supported process gains should not be assumed to translate directly into evaluated artistic outcomes. Prior studies indicate that the relationship between technology-supported learning processes and performance outcomes is not necessarily linear (He et al., 2025). Research suggests that perceived ease and efficiency may create illusions of learning, where individuals overestimate their understanding without corresponding improvements in performance (Bjork et al., 2013). The concept of cognitive offloading further highlights that reliance on external tools may reduce cognitive effort and limit opportunities for internalization (Risko and Gilbert, 2016). Emerging evidence suggests that AI tools may reinforce these tendencies, particularly in technology-rich learning environments (Gerlich, 2025). This does not mean that offloading is always harmful. In creative work, external support can free attention for higher-level judgment, comparison, and revision. The issue is whether students use that support to deepen creative decision-making or to bypass it. These findings provide a theoretical basis for examining process–outcome decoupling, in which stronger perceived support or engagement does not necessarily correspond to stronger assessed performance.

This issue is closely related to sustainable learning, which emphasizes long-term capability development rather than short-term performance gains (Ansari, 2025). In creative disciplines, sustainable learning means more than producing a polished final image. It involves transferable artistic judgment, independent decision-making, the ability to explain creative choices, and the capacity to continue learning beyond a single course task. Self-regulated learning perspectives are useful here because they emphasize planning, monitoring, strategy use, reflection, and adaptation as central to meaningful learning (Zimmerman, 1990; Pintrich, 2000). GenAI may support development-oriented learning when it helps students compare alternatives, clarify intentions, revise decisions, and reflect on process. It may work against such learning when students become dependent on generated outputs without developing the ability to explain, adapt, or internalize creative decisions. The educational question is therefore not simply whether AI is used, but whether AI-supported activity becomes part of students' own creative agency.

Taken together, the existing literature remains theoretically and empirically insufficient in several important respects. First, many studies examine AI adoption, frequency, or perceived usefulness without distinguishing the role AI plays in the learning process. Second, research on GenAI and creativity has provided important evidence on idea generation and creative output, but less evidence on visible authorship, internalization, and assessment in studio-based education. Third, studies based mainly on perceptions or short-term tasks provide limited insight into how AI-supported learning relates to prior ability and evaluated performance. Finally, the relationship between AI-supported process experience and development-oriented learning remains underexplored. These gaps are especially relevant for creative education because process support, assessed artistic performance, visible authorship, and longer-term capability may not move in the same direction. A differentiated analytical approach is therefore needed, one that examines AI use not only as adoption or intensity, but also as a mode of participation in the creative process.

## 3 Methodology

To address this limitation, this study proposes a differentiated analytical framework that distinguishes between assistive AI usage and content-generative AI usage, and examines how these two modes of engagement relate to artistic outcomes, learning processes, and development-oriented outcomes. The analysis draws on linked course data, including students' prior course performance, final creative-performance assessment, and questionnaire responses on AI-supported creative learning. This design allows AI use to be examined in relation to both students' earlier performance levels and their final creative outcomes.

The overall analytical strategy is organized around four research questions. The sequence moves from artistic outcomes to learning experience, psychological interpretation, and longer-term developmental orientation.

- **RQ1:** How are different modes of AI use, specifically assistive use and content-generative use, associated with multiple dimensions of artistic outcomes, including creativity, aesthetic quality, technique, authorship, completion, and overall performance?
- **RQ2:** How are these differentiated AI usage patterns associated with learning-related constructs, such as perceived support, creative experience, skill transfer, and agency?
- **RQ3:** How is AI use associated with students' psychological perceptions, including creative confidence and presentation readiness, and how do these factors relate to artistic outcomes?
- **RQ4:** How are differentiated AI usage patterns associated with development-oriented learning outcomes, including continuation intention, institutional collaboration, social value orientation, and future development orientation?

To address these research questions, this study uses a course-based analytical design that links prior course performance, final creative-performance evaluation, and questionnaire-based measures of students' creative learning processes. The empirical analysis first examines differences in AI usage patterns and artistic outcomes, and then introduces prior course performance to evaluate whether the observed patterns remain stable across students with different earlier performance levels. Performance-change analysis is used to compare final assessment with prior course performance, while additional robustness checks examine the consistency of the findings across different definitions and intensities of AI use. The methodological details are presented in the following subsections.

### 3.1 Data collection

This study was conducted with the approval of Communication University of Zhejiang. All participants were informed about the purpose of the study and provided informed consent before data collection. Participation was voluntary, and all questionnaire and performance data were processed using de-identified student codes. These codes allowed questionnaire responses to be linked with prior course performance and final artistic evaluations while preventing students' personal identities from entering the analytical dataset. Students were told that their questionnaire responses would not affect course grading, in order to reduce potential response bias (Zawacki-Richter et al., 2019). These procedures are consistent with established ethical guidelines in technology-enhanced learning research (Holmes et al., 2022).

Data were collected from three linked sources: students' prior course performance records, final artistic performance evaluations, and a structured questionnaire on AI usage patterns, learning processes, and psychological perceptions. The prior performance records provided a baseline for students' earlier course achievement, while the final evaluation captured the quality of the completed creative work. The questionnaire was used to document how students engaged with AI during the creative process and how they perceived its role in their learning.

#### 3.1.1 Educational context and participants

The study was conducted within two art-related undergraduate courses: Character Design and Animation Concept Design. Four intact undergraduate classes participated in the study. The four classes included 151 students in total, with class sizes of 41, 32, 38, and 40 students, respectively. The two third-year classes were from the animation major and were enrolled in Animation Concept Design. The first-year and second-year classes were from the illustration and comic art track and were enrolled in Character Design. All students in these classes were invited because the study was embedded in regular course-based creative projects rather than conducted as an externally assigned experiment. After removing one duplicated questionnaire submission with identical responses from the same student, 136 non-duplicated questionnaire responses were retained. Three responses were excluded because they were incomplete and lacked key analytical variables. The questionnaire response rate after removing the duplicated submission was 90.1% (136/151), and the final valid-case rate was 88.1% of all students recorded in the course score sheets (133/151) and 97.8% of the non-duplicated questionnaire responses (133/136). The final sample of 133 valid cases was considered appropriate for the planned baseline-adjusted regression models and supplementary robustness checks, while the single-institution and course-based sampling design was considered when interpreting the findings.

The final valid sample included 38 first-year students (28.6%), 34 second-year students (25.6%), and 61 third-year students (45.9%). In terms of gender, 25 students identified as male (18.8%), 96 as female (72.2%), and 12 preferred not to disclose their gender (9.0%). The sample included 61 students from the animation major (45.9%) and 72 students from the illustration and comic art track (54.1%). No valid case had missing grade, gender, or major information. All creative tasks were designed under a unified thematic direction aligned with curriculum-based cultural and educational objectives, focusing on traditional cultural topics such as intangible cultural heritage and historical artifacts. This design ensured consistency across student projects while allowing flexibility in creative expression. Such structured yet open-ended task design is consistent with prior research on creative learning environments and creativity-oriented learning design (Zawacki-Richter et al., 2019; Chen et al., 2020). This context provides a natural course setting for examining AI-supported creative learning, including students' creative processes, final artistic outcomes, and patterns of AI use.

#### 3.1.2 Performance evaluation data

Students' artistic performance was assessed at two stages. First, prior course performance was recorded through daily performance (*daily_score*) and mid-term evaluation (*mid_score*), which were used to describe students' earlier learning status before the final project assessment. Second, final artistic outcomes were evaluated across five dimensions: creativity, aesthetic quality, technique, authorship, and completion. Each dimension was scored on a 20-point scale, resulting in a maximum total score of 100. A composite score (*final_total*) was calculated as the sum of the five dimensions. The five evaluation dimensions are defined as follows:

- **Creativity**: originality and innovativeness of the concept.
- **Aesthetic quality**: visual appeal, composition, and stylistic coherence.
- **Technique**: level of technical execution and craftsmanship.
- **Authorship**: visible evidence of independent creative intention, decision-making, and personal ownership as expressed in the final artifact.
- **Completion**: completeness and refinement of the work.

To ensure reliability, two full-time instructors with doctoral degrees in relevant disciplines and more than 7 years of teaching experience independently evaluated all works. The evaluators received only the submitted artworks and the standardized scoring rubric during assessment. They did not have access to students' questionnaire responses, AI-use status, or AI-use percentage variables. This procedure was used to reduce the possibility that AI-use information would influence artistic scoring.

A standardized scoring rubric was established prior to evaluation. Creativity assessed originality, conceptual development, and the distinctiveness of the artistic idea. Aesthetic quality assessed visual appeal, composition, stylistic coherence, and expressive consistency. Technique assessed technical execution, craftsmanship, and control of the chosen medium or digital process. Authorship assessed visible evidence of independent creative intention, decision-making, personal expression, and ownership in the final artifact. Completion assessed the completeness, refinement, and presentation quality of the submitted work.

Inter-rater agreement was examined before score reconciliation. Because the five dimensions were scored on ordered 20-point educational rating scales, agreement was assessed using Cohen's kappa after converting score differences between the two evaluators into predefined agreement bands. This approach provided a practical way to evaluate whether the two evaluators assigned substantively consistent scores across the five dimensions. The resulting kappa value was 0.92, indicating almost perfect agreement between the two evaluators before score reconciliation (Landis and Koch, 1977). Given the subjective nature of artistic evaluation, a structured reconciliation protocol was adopted. When the difference between the two evaluators' scores did not exceed 5%, the final score was calculated as their average. When the difference exceeded 5%, a third evaluator provided an additional assessment, and the final score was computed as the average of the two closest scores. All cases resulted in valid scores under this procedure. This multidimensional evaluation approach aligns with established frameworks for assessing creative performance in educational contexts (Runco and Beghetto, 2019).

#### 3.1.3 Questionnaire data

A structured questionnaire was administered after the completion of the final project to capture students' experiences during the creative process. The questionnaire was developed by the research team according to the research questions, the course context, and prior literature on AIED, creative learning, learner agency, skill transfer, authorship, and development-oriented learning (Zawacki-Richter et al., 2019). Because the questionnaire was designed for this specific course-based study, the items were not taken directly from a single existing standardized scale. Instead, items were constructed to represent the theoretical dimensions examined in the study and were aligned with the analytical framework distinguishing assistive AI use, content-generative AI use, learning processes, psychological perceptions, and development-oriented outcomes. To strengthen content validity, the questionnaire was reviewed before administration by two senior researchers with extensive experience in art education and by one researcher specializing in AIED. The reviewers examined whether the items were understandable to undergraduate art students, whether they corresponded to the intended constructs, and whether the questionnaire covered the key dimensions of AI-supported creative learning. Revisions were made to improve wording clarity and construct alignment before data collection.

To reduce possible comprehension problems, students were informed before completing the questionnaire that they could contact a member of the research team who was not their course instructor if any item was unclear. The instructional team also explained the distinction between AI as process support and AI as content contribution before questionnaire completion. No clarification requests were received before the questionnaire collection was completed. The validity argument for the questionnaire followed a construct-oriented approach, in which validity is understood as evidence supporting the interpretation of scores rather than as a property established by a single statistic (Messick, 1995). Evidence related to construct validity was considered at three levels. First, item development was guided by the theoretical constructs and research questions. Second, the questionnaire was reviewed by domain experts before administration. Third, after data collection, the internal structure of the measures was examined through Cronbach's alpha and the correlation pattern among constructs. The correlation structure was inspected to determine whether theoretically related constructs showed meaningful associations while remaining empirically distinguishable from teacher-rated artistic performance. These procedures provided content-based and internal-structure evidence for the intended use of the questionnaire measures.

The questionnaire consisted of seven main components:

1. **Background information:** students reported demographic and academic characteristics, including grade level, major, prior experience with AI tools, and career intentions.
2. **AI usage patterns:** students reported whether AI tools were used, the types of tools used, and the stages of the creative process in which AI was involved. AI usage was operationalized through two key indicators: the proportion of AI-assisted support (*ai_help_pct*) and the proportion of AI-generated content (*ai_content_pct*). This operationalization reflects the distinction between augmentation-oriented and substitution-oriented AI usage, which is central to the analytical framework of this study (Lee et al., 2024).
3. **Modes of engagement with AI:** this section captured how AI was integrated into the creative workflow, including idea generation, process support, technical learning, reference acquisition, and direct content creation. These distinctions were used to describe the specific role of AI in students' creative work, from support for the creative process to direct contribution to creative output (Lee et al., 2024; Chen et al., 2020).
4. **Learning process constructs:** Likert-scale items (1–5) were used to measure perceived support, creative experience, aesthetic judgment, skill transfer, and learner agency. These constructs reflect key dimensions of learning processes emphasized in learning analytics research (Siemens, 2013; Márquez et al., 2024).
5. **Psychological variables:** students' perceptions of their creative abilities were measured through constructs such as creative confidence, presentation readiness, identity-related perceptions, and self-evaluation. These variables capture affective and cognitive aspects of learning that are critical for sustained engagement in creative education (Runco and Beghetto, 2019; Chen et al., 2020).
6. **Developmental and societal perspectives:** students reported their willingness for continued creative work, perceived social value of their work, and intentions for future development. These dimensions align with broader goals of sustainable education (Ansari, 2025; Holmes et al., 2022).
7. **Course and instructional evaluation:** students evaluated the design of the course and instructional approach, including clarity of learning objectives, task appropriateness, integration of AI tools, and teaching effectiveness. This component complements the variable *teaching_ai_climate* and provides a broader understanding of the instructional context (Zawacki-Richter et al., 2019).

The two percentage indicators, *ai_help_pct* and *ai_content_pct*, were based on students' self-estimates rather than system log data. To improve interpretability, the questionnaire provided explanations for the relevant AI-use items and asked students to report approximate proportions rather than exact measurements. *ai_help_pct* referred to the perceived proportion of AI assistance in the creative process, including ideation, reference search, revision, process organization, and technique learning. *ai_content_pct* referred to the perceived proportion of the final creative output that was directly generated or substantially shaped by AI-generated content. These variables were therefore interpreted as students' perceived AI involvement rather than precise behavioral measurements. This interpretation is appropriate for the present study because AI use in creative work often occurs across multiple stages and cannot always be objectively separated into discrete units. To reduce reliance on a single operationalization, the analysis also included alternative AI-use definitions based on reported tool types, creative stages, engagement modes, and non-zero AI-help or AI-content proportions.

In addition, open-ended questions were included to capture students' reflections on their creative process and the role of AI. These qualitative responses provide complementary evidence for interpreting the quantitative findings. The internal consistency of all multi-item constructs was assessed using Cronbach's alpha. The detailed results are reported in Section 4.1. The full questionnaire instrument is provided in the Supplementary material S1.

### 3.2 Analytical strategy

The analysis proceeded in four steps. It first examined artistic performance, then learning-process variables, psychological perceptions, and development-oriented outcomes. Descriptive statistics were reported at the beginning to show the distribution of the main variables and to identify possible ceiling effects, skewed variables, or outliers.

For RQ1, the main outcome models examined whether different forms of AI use were related to final artistic performance. Since the dataset contained both earlier course performance and final creative-performance assessment, students' prior performance was included in the models. A baseline performance score was calculated from daily performance and mid-term evaluation using Equation (1):


$$\begin{array}{l} {\text{baseline\_score}}_{i}=\frac{{\text{daily\_score}}_{i}+{\text{mid\_score}}_{i}}{2}. \end{array}$$ (1)

This score was used to account for differences in students' earlier course achievement before the final project. The baseline-adjusted model is specified in Equation (2):


$$\begin{array}{l} Y_{i}^{\left(k\right)}=\beta_{0}^{\left(k\right)}+\beta_{1}^{\left(k\right)}{\text{ai\_use}}_{i}+\beta_{2}^{\left(k\right)}{\text{ai\_help\_pct}}_{i}+\beta_{3}^{\left(k\right)}{\text{ai\_content\_pct}}_{i}\\ +\beta_{4}^{\left(k\right)}{\text{baseline\_score}}_{i}+{\text{X}}_{i}\gamma^{\left(k\right)}+\varepsilon_{i}^{\left(k\right)}, \end{array}$$ (2)

where *i* indexes individual students and *k* denotes the dimension of artistic performance. The dependent variable $Y_{i}^{\left(k\right)}$ represents the corresponding final performance outcome, including creativity, aesthetic quality, technique, authorship, completion, and total score. **X**_*i*_ denotes a vector of control variables, including prior AI experience and relevant background characteristics. $\beta_{0}^{\left(k\right)}$ is the intercept, $\beta_{1}^{\left(k\right)}$–$\beta_{4}^{\left(k\right)}$ are coefficients for AI usage and baseline performance variables, **γ**^(*k*)^ represents coefficients for control variables, and $\varepsilon_{i}^{\left(k\right)}$ is the error term. Because students came from different year-level cohorts, courses, and majors, these differences were addressed in three ways: prior course performance was included in the main baseline-adjusted models; grade, major, gender, and work type were included in the propensity-score model; and key models were re-estimated with grade fixed effects as a sensitivity check.

A performance-change variable was also constructed using Equation (3):


$$\begin{array}{l} {\text{performance\_change}}_{i}={\text{final\_total}}_{i}-{\text{baseline\_score}}_{i}. \end{array}$$ (3)

The corresponding performance-change model is specified in Equation (4):


$$\begin{array}{l} {\text{performance\_change}}_{i}=\pi_{0}+\pi_{1}{\text{ai\_use}}_{i}+\pi_{2}{\text{ai\_help\_pct}}_{i}\\ \;+\pi_{3}{\text{ai\_content\_pct}}_{i}+\pi_{4}{\text{baseline\_score}}_{i}\\ \;+{\text{X}}_{i}\omega +u_{i}. \end{array}$$ (4)

This model served as a supplementary check of baseline-to-final change. Because the baseline and final scores came from related but not identical course assessments, *performance_change* was treated as a supplementary indicator rather than a direct experimental gain score. The hypotheses for RQ1 are as follows:

- H1a: AI usage is associated with overall artistic performance, with heterogeneous patterns across different performance dimensions.
- H1b: Assistive AI usage is positively associated with technical execution and aesthetic quality, and may also relate to other performance dimensions, including creativity, authorship, and completion.
- H1c: Content-generative AI usage is negatively associated with authorship and originality-related outcomes, and exhibits heterogeneous associations across other performance dimensions.

Several supplementary checks were added to the RQ1 analysis. Quadratic terms of AI usage variables were included to examine possible non-linear patterns, and AI usage intensity was grouped into multiple levels for comparison. Multivariate analysis of variance (MANOVA) was used to evaluate joint patterns across multiple performance dimensions. False discovery rate (FDR) correction was applied to control for multiple comparisons and reduce the likelihood of Type I errors (Benjamini and Hochberg, 1995).

Propensity-score analyses were also conducted to compare AI users and non-users with similar observed characteristics. The primary treatment indicator was based on the questionnaire item indicating whether students used AI tools in the project. Propensity scores were estimated using logistic regression with daily score, mid-term score, prior AI experience, grade, major, gender, and work type as covariates. AI users were matched to non-users using 1:1 nearest-neighbor matching on the estimated propensity score, with replacement and without a caliper. Replacement was allowed because the number of non-users was smaller than the number of AI users and because several control cases provided the closest matches for multiple treated cases. A caliper sensitivity check using 0.2 standard deviations of the logit propensity score was also examined, but it did not improve covariate balance relative to the main nearest-neighbor specification. Common support was assessed by comparing the propensity-score ranges of AI users and non-users. Covariate balance was evaluated using standardized mean differences before and after matching. Inverse-probability weighting (IPW) was used as a second propensity-score adjustment approach. Propensity scores were clipped to the interval (0.05, 0.95) to reduce instability from extreme weights. Stabilized weights were inspected as a diagnostic, including the mean, maximum, and effective sample size of the weights. The PSM and IPW analyses were applied to the main performance outcomes and key learning-process variables, including final total score, performance change, authorship, perceived support, skill transfer, and agency.

Robustness checks were further conducted using alternative operational definitions of AI use. The main definition was based on the questionnaire item on AI use. Additional definitions were constructed from reported AI tool types, creative stages, engagement modes, and non-zero AI-help or AI-content proportions. The key models were repeated across these definitions to examine whether the main findings remained stable when AI use was coded in different ways.

Because students were taught within intact year-level course cohorts, possible group-level dependence was considered through grade-based sensitivity analysis. Grade was used as the grouping variable because it represented the intact course-cohort structure in the available dataset and captured the main differences in year level, course stage, disciplinary track, and instructional context. A full multilevel model was not estimated because the study involved only a small number of intact course cohorts, which would make multilevel variance estimates and cluster-robust standard errors unstable. Therefore, the main analyses used HC3 robust standard errors, and key models were re-estimated with grade fixed effects as a sensitivity check. For multiple testing, Benjamini–Hochberg false discovery rate (FDR) correction was applied within each family of related regression tests, and adjusted *p*-values were reported in the corresponding result tables. Results were interpreted by considering unadjusted *p*-values, FDR-adjusted *p*-values, effect direction, confidence intervals, and consistency across robustness checks. Because some performance outcomes showed skewness or upper-range concentration, OLS results were interpreted together with distributional diagnostics. When distributional features raised modeling concerns, additional sensitivity analyses were conducted using a binary below-maximum authorship model and rank-transformed authorship models. These analyses examined whether the observed authorship patterns depended on the raw-score OLS specification.

For RQ2, the analysis examined learning process variables. The relationship between AI usage and learning variables is modeled in Equation (5):


$$\begin{array}{l} L_{i}^{\left(m\right)}=\alpha_{0}^{\left(m\right)}+\alpha_{1}^{\left(m\right)}{\text{ai\_use}}_{i}+\alpha_{2}^{\left(m\right)}{\text{ai\_help\_pct}}_{i}+\alpha_{3}^{\left(m\right)}{\text{ai\_content\_pct}}_{i}\\ +{\text{X}}_{i}\delta^{\left(m\right)}+\varepsilon_{i}^{\left(m\right)}, \end{array}$$ (5)

where $L_{i}^{\left(m\right)}$ denotes the learning process outcome for student *i* in dimension *m*, including perceived support, creative experience, aesthetic judgment, skill transfer, and learner agency. Other variables are defined as previously described. The hypotheses for RQ2 are as follows:

- H2a: Assistive AI usage is positively associated with learning support, engagement, and skill transfer, and is also associated with broader learning process variables, including creative experience, aesthetic judgment, and learner agency.
- H2b: Content-generative AI usage is negatively associated with learner agency and is associated with other learning process variables related to active engagement.
- H2c: Learning process variables are positively associated with artistic performance across multiple dimensions.
- H2d: Learning process variables show pathway-consistent relationships between AI usage and artistic performance.

Learning process variables were then incorporated into performance models, as specified in Equation (6):


$$\begin{array}{l} Y_{i}^{\left(k\right)}=\lambda_{0}^{\left(k\right)}+\lambda_{1}^{\left(k\right)}L_{i}^{\left(m\right)}+{\text{X}}_{i}\kappa^{\left(k\right)}+\varepsilon_{i}^{\left(k\right)}, \end{array}$$ (6)

where *i* indexes individual students, *k* denotes the *k*-th dimension of artistic performance, and *m* denotes the *m*-th learning process dimension. The dependent variable $Y_{i}^{\left(k\right)}$ represents the corresponding performance outcome, and $L_{i}^{\left(m\right)}$ denotes the learning process variable. **X**_*i*_ is a vector of control variables as defined previously. $\lambda_{0}^{\left(k\right)}$ is the intercept term, $\lambda_{1}^{\left(k\right)}$ captures the relationship between the learning process variable and the outcome, **κ**^(*k*)^ is a vector of coefficients corresponding to **X**_*i*_, and $\varepsilon_{i}^{\left(k\right)}$ is the error term.

Pathway analysis was conducted using a regression-based framework following established approaches in mediation analysis (Baron and Kenny, 1986; Igartua and Hayes, 2021). These models were used to examine whether the observed associations were statistically consistent with the proposed pathway, rather than to identify causal mediation. The intermediate-variable and outcome models are specified in Equations (7) and (8), respectively:


$$\begin{array}{l} M_{i}=a_{0}+a_{1}{\text{ai\_help\_pct}}_{i}+a_{2}{\text{ai\_content\_pct}}_{i}+{\text{X}}_{i}\alpha +\varepsilon_{i}, \end{array}$$ (7)

$$\begin{array}{l} Y_{i}=c_{0}^{\prime }+c_{1}^{\prime }{\text{ai\_help\_pct}}_{i}+c_{2}^{\prime }{\text{ai\_content\_pct}}_{i}+bM_{i}+{\text{X}}_{i}\beta +\varepsilon_{i}, \end{array}$$ (8)

where *M*_*i*_ denotes the intermediate variable capturing learning process or psychological perception, and *Y*_*i*_ represents the outcome variable. The coefficients *a*_1_ and *a*_2_ capture the relationships between AI usage variables and the intermediate variable, while *b* captures the relationship between the intermediate variable and the outcome. The indirect pathway is quantified as the product *a* × *b*, and its statistical stability is assessed using bootstrap resampling. Other variables are defined as previously described.

For RQ3, the analysis focused on psychological perceptions. The relationship between AI usage and psychological variables is modeled in Equation (9):


$$\begin{array}{l} P_{i}^{\left(n\right)}=\theta_{0}^{\left(n\right)}+\theta_{1}^{\left(n\right)}{\text{ai\_use}}_{i}+\theta_{2}^{\left(n\right)}{\text{ai\_help\_pct}}_{i}+\theta_{3}^{\left(n\right)}{\text{ai\_content\_pct}}_{i}\\ +{\text{X}}_{i}\eta^{\left(n\right)}+\varepsilon_{i}^{\left(n\right)}, \end{array}$$ (9)

where $P_{i}^{\left(n\right)}$ denotes the psychological outcome for student *i* in dimension *n*, including creative confidence, presentation readiness, and identity-related constructs. Other variables are defined as previously described. The hypotheses for RQ3 are as follows:

- H3a: AI usage is positively associated with creative confidence and self-evaluation, and is also associated with broader psychological variables, including presentation readiness and creative identity.
- H3b: Psychological variables are positively associated with artistic performance outcomes across multiple dimensions.
- H3c: Content-generative AI usage is negatively associated with psychological ownership and creative identity, and is associated with broader psychological engagement.
- H3d: Psychological variables show pathway-consistent relationships between AI usage and artistic performance.

Psychological variables were then incorporated into performance models, as specified in Equation (10):


$$\begin{array}{l} Y_{i}^{\left(k\right)}=\lambda_{0}^{\left(k\right)}+\lambda_{1}^{\left(k\right)}P_{i}^{\left(n\right)}+{\text{X}}_{i}\kappa^{\left(k\right)}+\varepsilon_{i}^{\left(k\right)}. \end{array}$$ (10)

To examine learning and psychological variables together, extended models were estimated as specified in Equation (11):


$$\begin{array}{l} Y_{i}^{\left(k\right)}=\lambda_{0}^{\left(k\right)}+\lambda_{1}^{\left(k\right)}L_{i}^{\left(m\right)}+\lambda_{2}^{\left(k\right)}P_{i}^{\left(n\right)}+{\text{X}}_{i}\kappa^{\left(k\right)}+\varepsilon_{i}^{\left(k\right)}. \end{array}$$ (11)

Finally, RQ4 examined development-oriented outcomes. The corresponding model is specified in Equation (12):


$$\begin{array}{c} D_{i}^{\left(s\right)}=\phi_{0}^{\left(s\right)}+\phi_{1}^{\left(s\right)}{\text{ai\_use}}_{i}+\phi_{2}^{\left(s\right)}{\text{ai\_help\_pct}}_{i}\\ +\phi_{3}^{\left(s\right)}{\text{ai\_content\_pct}}_{i}+\phi_{4}^{\left(s\right)}{\text{teaching\_ai\_climate}}_{i}\\ +\lambda_{1}^{\left(s\right)}L_{i}^{\left(m\right)}+\lambda_{2}^{\left(s\right)}P_{i}^{\left(n\right)}\\ +{\text{X}}_{i}\psi^{\left(s\right)}+\varepsilon_{i}^{\left(s\right)}. \end{array}$$ (12)

where $D_{i}^{\left(s\right)}$ denotes development-oriented outcomes, including continuation intention, institutional collaboration, and perceived social value. Other variables are defined as previously described. The hypotheses for RQ4 are as follows:

- H4a: Assistive AI usage is positively associated with development-oriented outcomes, including continuation intention and perceived social value, and is also associated with broader outcomes such as institutional collaboration.
- H4b: Content-generative AI usage exhibits heterogeneous associations with development-oriented outcomes across different dimensions.
- H4c: Content-generative AI usage does not show a stable direct positive association with development-oriented outcomes after accounting for internalization and teaching AI climate.

Subsample analyses restricted to AI users were conducted, and robustness checks included non-linear specifications, alternative variable constructions, and alternative definitions of AI use. Additional models examined the relationship between AI usage and instructional AI climate. Across the main regression analyses, ordinary least squares (OLS) estimation with HC3 robust standard errors was applied to address potential heteroskedasticity (White, 1980; MacKinnon and White, 1985).

## 4 Results and discussion

This section presents the empirical findings and discusses them in relation to the proposed analytical framework. The analysis focuses on how different forms of AI use relate to creative learning processes, artistic performance, and development-oriented outcomes. We begin by examining the data structure and assessing the reliability of the questionnaire-based measures. The results are then organized according to the four research questions. First, we investigate the relationship between AI usage and multidimensional artistic performance, including baseline-adjusted and performance-change analyses (RQ1). Second, we examine how AI usage is related to learning process variables such as perceived support, creative experience, skill transfer, and agency (RQ2). Third, we analyze psychological factors and their links with artistic performance (RQ3). Finally, we explore development-oriented outcomes, including continuation intention, institutional collaboration, and perceived social value (RQ4).

This sequence brings together performance outcomes, learning processes, and psychological variables in the same results section. It also allows the process–outcome decoupling pattern to be examined across different levels of evidence, including final performance, baseline-to-final change, process-related questionnaire measures, and supplementary robustness checks.

### 4.1 Data structure and instrument reliability

The final analytical dataset included 133 matched cases, linking students' prior course performance, final artistic evaluation, and questionnaire responses. Table 1 reports the descriptive statistics of the main variables. Both prior and final performance scores were concentrated in the upper range. The baseline score had a mean of 89.511 and a median of 92.00, while the final total score had a mean of 87.677 and a median of 89.00. The baseline-to-final change score had a mean of −1.835 and a median of −1.50. In this section, the change score is used as a course-based indicator of movement from earlier performance to final assessment.

**Table 1:** Descriptive statistics of main variables.

Variable	*N*	Mean	Median	SD	Min	Max	Skew.	Kurt.
Performance outcomes								
Baseline score	133	89.511	92.00	9.006	45.00	98.50	−2.873	9.470
Final total	133	87.677	89.00	7.970	61.00	99.00	−1.084	0.906
Performance change	133	−1.835	−1.50	8.766	−25.50	32.00	0.444	2.462
Creativity	133	18.308	19.00	1.207	14.00	20.00	−1.179	1.480
Aesthetic quality	133	17.256	17.00	1.704	12.00	20.00	−0.652	0.251
Technique	133	16.323	17.00	2.102	12.00	20.00	−0.599	−0.475
Authorship	133	18.842	20.00	2.160	9.00	20.00	−2.771	7.979
Completion	133	16.947	17.00	2.086	12.00	20.00	−0.678	−0.352
Learning and psychological constructs								
Support	133	3.502	3.60	0.706	1.00	5.00	−1.235	3.026
Creative experience	133	3.483	3.50	0.755	1.00	5.00	−0.572	1.386
Aesthetic judgment	133	3.998	4.00	0.684	1.00	5.00	−0.755	1.868
Skill transfer	133	3.735	3.75	0.619	1.00	5.00	−0.583	2.245
Agency	133	3.971	4.00	0.554	1.889	5.00	−0.423	0.493
Quality confidence	133	3.606	3.60	0.745	1.60	5.00	−0.054	0.072

The upper-range concentration of performance scores is not only a statistical feature of the dataset. In studio-based creative courses, high completion and high rubric scores often indicate that most students have met the formal requirements of the task. The more important question is how AI use relates to the specific dimensions through which artistic learning is recognized. Authorship had the highest mean (18.842) and a median of 20.00, with strong negative skewness (−2.771) and high kurtosis (7.979). Technique and completion showed wider dispersion, with standard deviations above 2.00 and lower kurtosis. These patterns show that the total score and some dimensions are concentrated near the upper range, while other dimensions retain stronger discriminative power. This is consistent with multidimensional approaches to creative-performance assessment, where aggregate scores and dimension-level ratings capture different aspects of artistic work (Runco and Beghetto, 2019).

Figure 1 visualizes this structure. Figure 1A shows that both baseline and final total scores are concentrated in the upper score range. Figure 1B separates the five final performance dimensions on the same 0–20 scale, making clear that authorship is highly concentrated near the maximum score, while technique and completion contain more variation. Figure 1C shows that performance change is centered close to zero, with the mean change at −1.83. This pattern gives a basis for the later performance models. Final total score captures overall artistic achievement, but it does not fully show how AI use may relate to particular forms of artistic competence. Dimension-level analysis is therefore necessary, especially for authorship, where small differences carry educational meaning because the construct is tied to independent intention and ownership. The distributional pattern also points to the process–outcome decoupling problem: stronger learning-process experience should not be assumed to appear automatically as higher final total scores. This point is consistent with research on illusions of learning and cognitive offloading, which shows that perceived ease or support does not always translate into stronger performance (Bjork et al., 2013; Risko and Gilbert, 2016).

**Figure 1:**
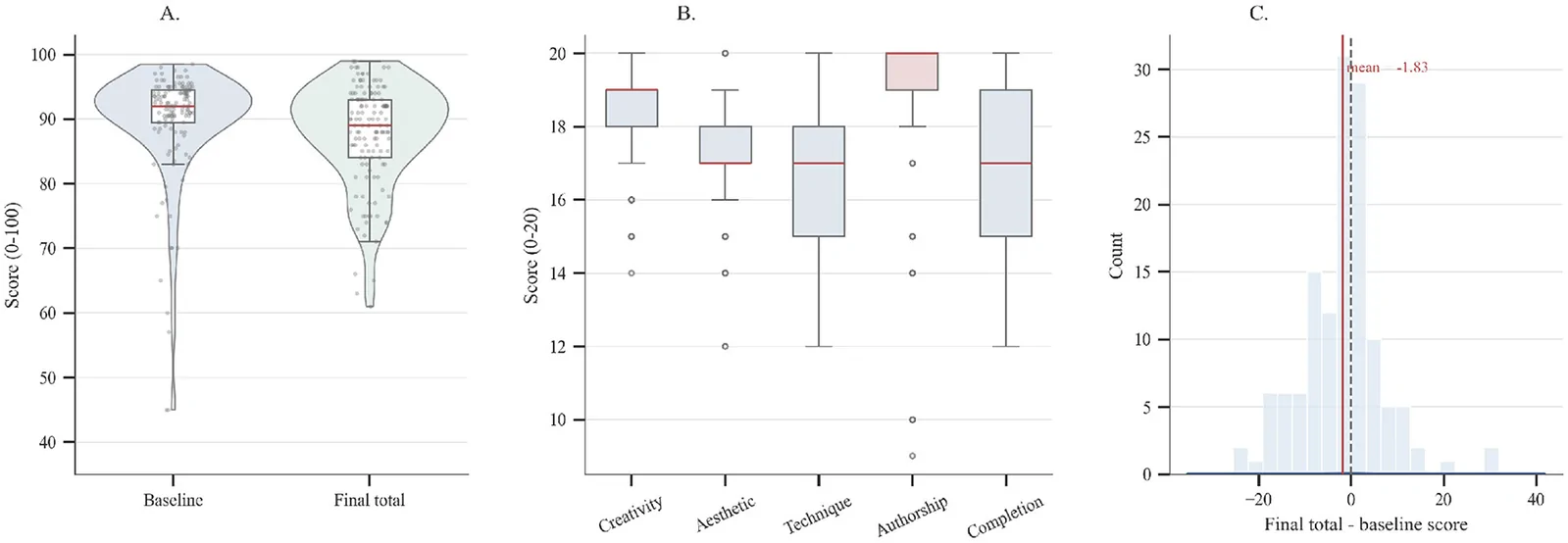
Performance profile across **(A)** baseline, final total, **(B)** final dimensions, and **(C)** baseline-to-final change.

The questionnaire constructs showed acceptable to strong internal consistency. As reported in Table 2, Cronbach's α values ranged from 0.740 for skill transfer to 0.945 for institutional collaboration. The core learning-process constructs all exceeded the commonly used 0.70 threshold: support (α = 0.814), creative experience (α = 0.911), aesthetic judgment (α = 0.902), skill transfer (α = 0.740), and agency (α = 0.781). The psychological and development-oriented constructs also showed strong reliability. These values support the use of the questionnaire measures in the subsequent learning-process, psychological, and development analyses. The reliability pattern is also consistent with established practice in learning analytics and educational measurement, where multi-item constructs are used to represent process-level learning experience (Siemens, 2013; Márquez et al., 2024).

**Table 2:** Internal consistency of questionnaire constructs.

Construct	Items	α	Mean	SD
Support	5	0.814	3.502	0.706
Creative experience	4	0.911	3.483	0.755
Aesthetic judgment	4	0.902	3.998	0.684
Skill transfer	4	0.740	3.735	0.619
Agency	9	0.781	3.971	0.554
Quality confidence	5	0.840	3.606	0.745
Display readiness	6	0.879	3.419	0.851
Continuation intention	4	0.943	3.793	0.854
Institutional collaboration	4	0.945	3.618	0.948
Social value orientation	5	0.905	3.687	0.790

Figure 2 presents the correlation structure of the main variables. The performance block shows strong internal coherence. Final total was strongly correlated with creativity (*r* = 0.87), aesthetic quality (*r* = 0.92), technique (*r* = 0.93), and completion (*r* = 0.93). Authorship was also positively related to final total (*r* = 0.68), but its correlations with other performance dimensions were lower, indicating that it captures a more distinct aspect of artistic evaluation. In contrast, the learning-process and psychological variables were only weakly related to final total. For example, support (*r* = 0.06), creative experience (*r* = −0.04), skill transfer (*r* = 0.03), and quality confidence (*r* = 0.02) showed little overlap with aggregate final performance.

**Figure 2:**
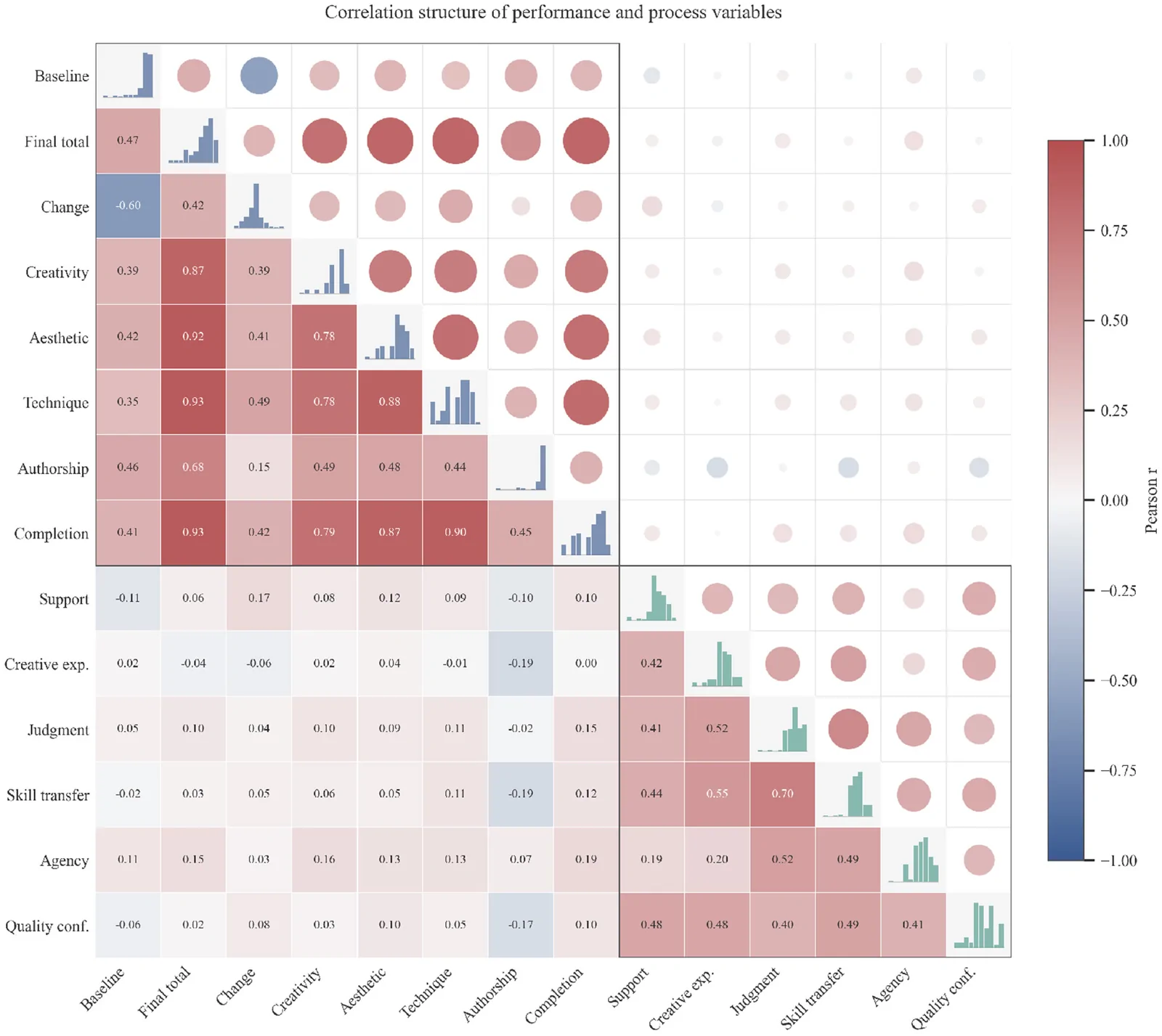
Correlation structure of performance outcomes, learning-process variables, and psychological constructs.

The weak correlations between learning-process constructs and final performance are theoretically important. They show that students' perceived support, creative experience, and skill transfer are not redundant with teacher-rated artistic quality. This separation supports treating process experience as a distinct layer of creative learning. AI may make the creative process more manageable, exploratory, or confidence-building, while final assessment remains tied to visible artistic quality, coherence, and authorship. Within the process layer, the constructs were meaningfully related to one another. Aesthetic judgment and skill transfer were strongly related (*r* = 0.70), and creative experience was moderately related to skill transfer (*r* = 0.55). These relationships indicate that the questionnaire captures a coherent process-level structure while remaining empirically separate from final performance evaluation.

AI usage variables were then examined before entering the regression models. Table 3 shows that students reported a higher average proportion of AI-assisted support (*ai_help_pct*: mean = 19.632, SD = 20.539) than AI-generated content in final outputs (*ai_content_pct*: mean = 12.158, SD = 17.412). The two variables were positively correlated (Pearson *r* = 0.729; Spearman *r* = 0.714), indicating that students who used AI for support often also used it for content generation. The VIF value for both variables was 3.178, which is below conventional thresholds for serious multicollinearity. The two indicators can therefore be retained together while still being treated as distinct dimensions of AI engagement.

**Table 3:** AI usage diagnostics and alternative AI-use definitions.

Indicator	*N*	Mean	SD	Min	Max	VIF
*ai_help_pct*	133	19.632	20.539	0	80	3.178
*ai_content_pct*	133	12.158	17.412	0	75	3.178
Additional diagnostics						
Pearson correlation	0.729					
Spearman correlation	0.714					
Mean of *ai_help_pct* + *ai_content_pct*	31.789					
Range of *ai_help_pct* + *ai_content_pct*	0–155					
Proportion above 100	6.02%					
AI-user counts under alternative definitions						
Main AI use: *ai_use* = 1	74					
Tool type not none	81					
Stage not none	82					
Mode not none	84					
Help or content > 0	92					

Figure 3 further clarifies the structure of the AI usage variables. Figure 3A shows a clear positive association between assistive and content-generative AI use, but the scatter also shows substantial dispersion. This confirms that the two variables overlap in practice while still preserving separate variation. Figure 3B shows that AI-user counts vary across operational definitions, from 74 under the main questionnaire-based definition to 92 when any non-zero AI-help or AI-content proportion is used. In creative work, AI use is rarely a single event. It may appear as tool selection, stage-specific assistance, workflow support, reference generation, or direct contribution to output. The different counts reflect how AI engagement appears through several traces in the creative process. This supports the decision to analyze both binary AI use and intensity-based measures, and to distinguish assistive use from content-generative use in the main models.

**Figure 3:**
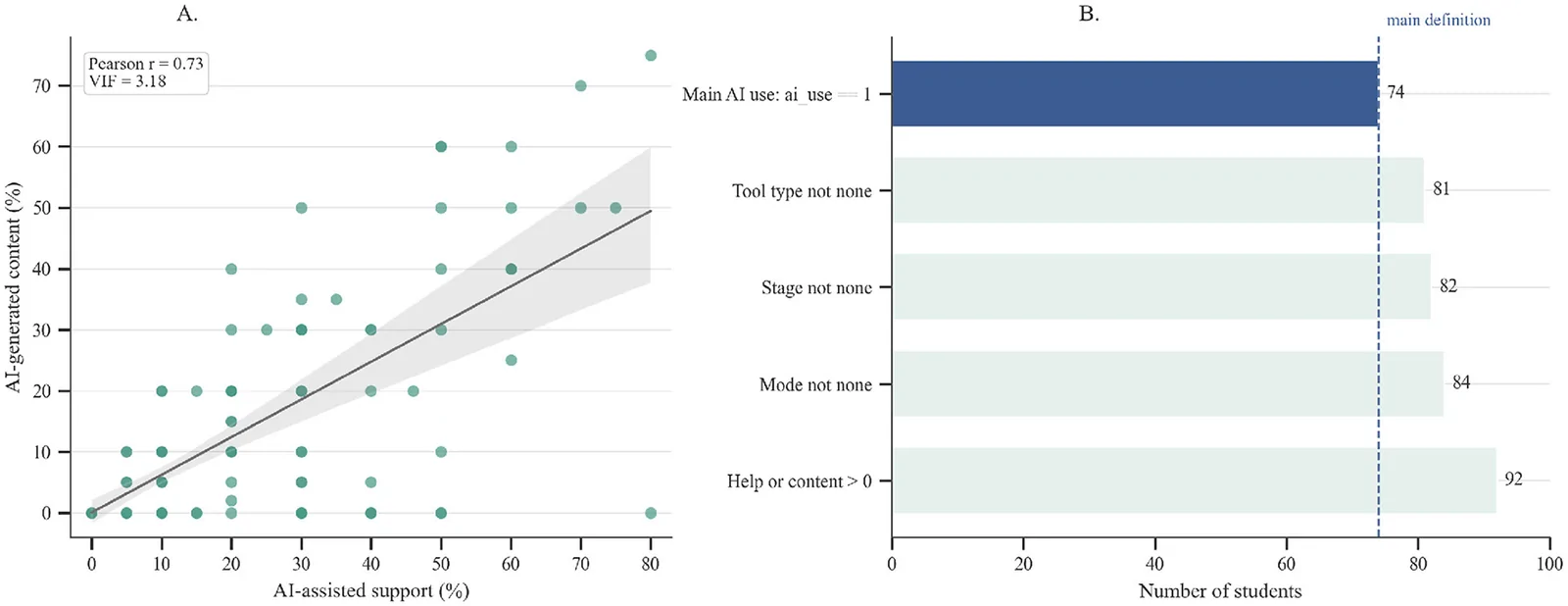
AI usage diagnostics based on usage intensity and alternative AI-user definitions. **(A)** Assistive and content-generative AI use. **(B)** AI-user counts by definition.

Together, these preliminary results point to the main tension examined in the study. The course produced generally strong final work, but the strongest variation lies in specific performance dimensions and in students' reported learning processes. The dataset therefore supports an analysis that moves beyond aggregate performance and examines how different forms of AI use relate to artistic outcomes, authorship, and process-level learning experience. The next subsection turns to this question by examining AI usage patterns under baseline-adjusted, dimension-level, and performance-change models.

### 4.2 AI usage, artistic performance, and learning mechanisms

The baseline-adjusted results show that AI use was not the main source of final-score differences in this course. As shown in Figure 4, prior course performance was the most stable predictor of final outcomes. Baseline performance was positively associated with final total score (β_*std*_ = 0.46, *p* < 0.001), creativity (β_*std*_ = 0.39, *p* < 0.001), aesthetic quality (β_*std*_ = 0.42, *p* < 0.001), technique (β_*std*_ = 0.35, *p* < 0.01), authorship (β_*std*_ = 0.37, *p* < 0.01), and completion (β_*std*_ = 0.43, *p* < 0.001). It was negatively associated with performance change (β_*std*_ = −0.61, *p* < 0.001), indicating that students with lower earlier scores had more room to improve by the final assessment. By contrast, binary AI use showed no stable association with final total score, performance change, or the five artistic dimensions. Assistive use and content-generative use followed the same pattern after baseline performance and prior AI experience were included.

**Figure 4:**
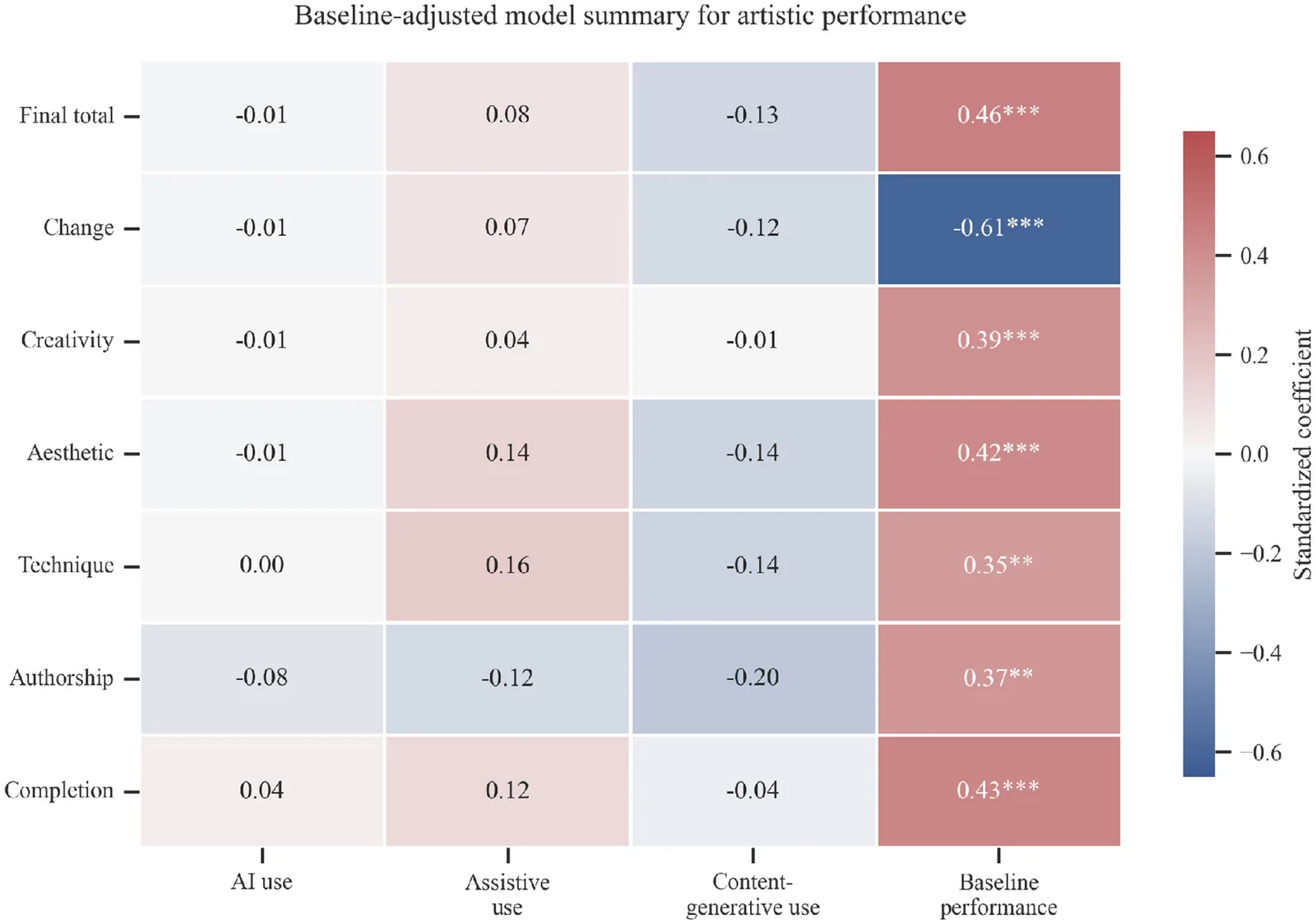
Baseline-adjusted model summary for artistic performance. Standardized coefficients are reported. ^**^p < 0.01, ^***^p < 0.001.

These results do not support the interpretation that AI use was associated with a broad final-score advantage in this course. After Benjamini–Hochberg FDR correction, the AI-use coefficients in the baseline-adjusted performance models did not remain significant at the 0.05 level. The baseline-adjusted coefficients are therefore interpreted as exploratory pattern evidence and are read together with the propensity-adjusted comparisons, dose profiles, and sensitivity analyses reported below. In a studio-based course, final work carries accumulated differences in visual judgment, drawing habits, technical fluency, and completion ability (Graham, 2019). AI may support parts of the workflow, but the present evidence does not indicate a corresponding overall performance advantage after earlier course performance was considered. H1a is therefore not supported as a broad final-score advantage of AI use. H1b also receives limited support in the baseline-adjusted performance models. The more consequential question is not whether AI was used, but what kind of work AI performed inside the creative process. This shift from tool adoption to mode of engagement is consistent with recent discussions of generative AI as a creative partner rather than a simple productivity device (Lee et al., 2024; Sabbaghan and Eaton, 2025; Creely and Blannin, 2025).

The propensity-adjusted comparisons provide the next layer of evidence. As reported in Table 4, AI users did not show a stable advantage in final total score or performance change. The PSM estimate for final total score was −1.58 with a 95% CI of (−4.30, 0.95), and the IPW estimate was −1.09 with a 95% CI of (−4.03, 2.06). A similar pattern appeared for performance change. In contrast, AI users reported higher perceived support and stronger skill transfer. The adjusted difference in support was 0.36 under PSM and 0.30 under IPW, while the adjusted difference in skill transfer was 0.49 under PSM and 0.38 under IPW. Agency also showed a positive pattern, especially under the PSM estimate. These findings shift the evidence from a performance-gain account to a process-gain account.

**Table 4:** Propensity-adjusted supplementary comparisons.

Outcome	PSM ATT	95% CI	IPW ATE	95% CI	Treated *N*	Matched *N*
Final total	−1.581	−4.297, 0.946	−1.094	−4.028, 2.063	74	30
Change	−1.196	−4.075, 1.473	0.129	−2.560, 2.877	74	30
Authorship	−1.270	−1.892, −0.743	−1.008	−1.648, −0.341	74	30
Support	0.365	0.170, 0.543	0.298	0.030, 0.585	74	30
Skill transfer	0.486	0.338, 0.642	0.378	0.105, 0.637	74	30
Agency	0.287	0.129, 0.443	0.173	−0.099, 0.431	74	30

PSM used 1:1 nearest-neighbor matching with replacement and no caliper. Matched N refers to unique matched controls. A caliper sensitivity check using 0.2 standard deviations of the logit propensity score was also examined, but it did not improve covariate balance relative to the no-caliper nearest-neighbor specification. The common-support interval was 0.2055–0.9286; one AI user and three non-users fell outside this interval. Matching reduced the mean absolute standardized mean difference from 0.185 to 0.130. IPW estimates used propensity scores clipped to (0.05, 0.95). Stabilized IPW diagnostics showed a mean weight of 1.02, a maximum weight of 6.22, and an effective sample size of 94.3. Estimates are interpreted as supplementary adjusted comparisons rather than causal effects.

These adjusted comparisons were accompanied by acceptable propensity-score diagnostics. The propensity-score model included daily score, mid-term score, prior AI experience, grade, major, gender, and work type, thereby aligning the adjusted comparisons with the baseline-linked design of the study. AI users were matched to non-users using 1:1 nearest-neighbor matching with replacement and without a caliper. The common-support interval was 0.2055–0.9286, with only one AI user and three non-users falling outside the interval. The mean matching distance was small (0.0079), indicating close matches on the estimated propensity score. Matching improved covariate balance, reducing the mean absolute standardized mean difference from 0.185 to 0.130 and the maximum absolute standardized mean difference from 0.530 to 0.246. The IPW diagnostics showed a similar level of stability: stabilized weights had a mean of 1.02, a maximum of 6.22, and an effective sample size of 94.3, indicating that the weighted estimates were not dominated by extreme weights. These diagnostics support using the propensity-adjusted results as supplementary robustness evidence for the process–outcome pattern, while the authorship dimension provides the clearest location where this pattern becomes evaluatively visible.

The same adjusted comparison identifies authorship as the most sensitive artistic dimension. AI users received lower evaluated authorship scores than non-users, and both adjustment strategies pointed in the same direction [PSM ATT = −1.27, 95% CI (−1.89, −0.74); IPW ATE = −1.01, 95% CI (−1.65, −0.34)]. This result gives partial support to H1c. The evidence is strongest when authorship is treated as a separate evaluative dimension rather than folded into the total score. AI use was associated with stronger process support and lower authorship evaluation at the same time. The observed AI-related pattern therefore appears as a redistribution of creative labor across exploration, technical support, production, and visible authorship. However, this pattern should not be interpreted as evidence that AI use directly caused lower authorship scores. The propensity-score analyses reduced observed differences between AI users and non-users, but they cannot rule out unmeasured confounding. The authorship pattern may also reflect differences in prior artistic habits, course-specific expectations, artwork type, or the extent to which AI-shaped visual features made students' own creative decisions less visible to evaluators.

The additional authorship sensitivity analyses support this interpretation. Because authorship was strongly concentrated near the upper end of the scale, logistic models were estimated for a binary below-maximum authorship outcome, and rank-transformed authorship models were estimated as an alternative to raw-score OLS. As reported in Table 5, AI use was associated with higher odds of receiving a below-maximum authorship score (OR = 4.67, FDR-adjusted *p* < 0.001). AI-help percentage and AI-generated-content percentage showed the same direction, with odds ratios of 1.038 and 1.051 per percentage point, respectively. Rank-transformed models also showed negative associations for AI use, AI-help percentage, and AI-generated-content percentage, all remaining significant after FDR correction. These sensitivity analyses suggest that the authorship pattern was unlikely to be an artifact of applying OLS to a ceiling-concentrated outcome.

**Table 5:** Authorship ceiling sensitivity analyses.

Model	Predictor	Coef.	OR	SE	*p*	FDR *p*
Below-max logit	AI use	1.542	4.672	0.408	<0.001	<0.001
Below-max logit	AI-help %	0.038	1.038	0.011	<0.001	<0.001
Below-max logit	AI-content %	0.049	1.051	0.014	<0.001	<0.001
Rank OLS	AI use	−24.881	–	5.475	<0.001	<0.001
Rank OLS	AI-help %	−0.563	–	0.138	<0.001	<0.001
Rank OLS	AI-content %	−0.772	–	0.169	<0.001	<0.001

Below-max authorship was coded as 1 when the authorship score was below 20 and 0 when it reached 20. Positive logit coefficients and odds ratios indicate higher odds of receiving a below-maximum authorship score. Rank OLS used rank-transformed authorship scores; negative coefficients indicate lower authorship ranks. FDR *p* values were adjusted using the Benjamini–Hochberg procedure.

The learning-process models provide the main regression evidence for H2. As shown in Table 6, binary AI use was positively associated with aesthetic judgment (β_*std*_ = 0.28, *p* = 0.010), skill transfer (β_*std*_ = 0.26, *p* = 0.010), and agency (β_*std*_ = 0.33, *p* = 0.004), with a marginal positive association for perceived support (β_*std*_ = 0.16, *p* = 0.055). These unadjusted results provide exploratory support for H2a: AI use was more clearly linked to students' learning-process experience than to their final artistic scores. However, after FDR correction, these coefficients did not remain significant at the 0.05 level. The learning-process results are therefore interpreted cautiously, with emphasis on their positive direction, consistency with the propensity-adjusted comparisons, and alignment with the descriptive dose profiles. H2b receives partial support. The separate AI-help and AI-content intensity coefficients were smaller and did not show stable independent associations in these models, which is consistent with the moderate overlap between the two forms of AI use in the dataset. This pattern also fits recent work on AIED and generative AI in higher education, where AI tools rarely operate as isolated interventions and more often combine guidance, feedback, exploration, and production (Lee et al., 2024; Sabbaghan and Eaton, 2025; Creely and Blannin, 2025).

**Table 6:** AI use and learning-process variables.

Outcome	Predictor	Coef.	SE	β_*std*_	*p*	FDR *p*
Support	AI use	0.223	0.116	0.157	0.055^†^	0.206
Support	AI-help %	0.003	0.006	0.088	0.596	0.734
Support	AI-content %	0.006	0.006	0.160	0.248	0.532
Creative experience	AI use	0.151	0.151	0.099	0.319	0.542
Creative experience	AI-help %	0.004	0.005	0.118	0.356	0.542
Creative experience	AI-content %	0.006	0.005	0.137	0.229	0.532
Aesthetic judgment	AI use	0.388	0.151	0.283	0.010^*^	0.051
Aesthetic judgment	AI-help %	−0.002	0.004	−0.059	0.636	0.734
Aesthetic judgment	AI-content %	0.001	0.004	0.037	0.740	0.793
Skill transfer	AI use	0.320	0.124	0.258	0.010^*^	0.051
Skill transfer	AI-help %	0.003	0.004	0.110	0.361	0.542
Skill transfer	AI-content %	0.003	0.004	0.071	0.530	0.723
Agency	AI use	0.371	0.130	0.334	0.004^**^	0.051
Agency	AI-help %	−0.000	0.004	−0.012	0.929	0.929
Agency	AI-content %	−0.005	0.004	−0.154	0.179	0.532

Coefficients are from HC3 robust OLS models controlling for baseline performance and prior AI experience. FDR *p* values were adjusted using the Benjamini–Hochberg procedure within this model family. None of the coefficients remained significant at the 0.05 level after FDR correction.

^†^*p* < 0.10, ^*^p < 0.05, ^**^p < 0.01.

The content-generative dose profile shows why the distinction between using AI and using AI for visible content matters. Figure 5 groups students by the reported proportion of AI-generated content in their work. Authorship declined from 19.38 in the no-content group to 19.00 in the 1%–20% group and 17.30 in the group above 20%. At the same time, perceived support increased from 3.28 to 3.53 and 4.00, while skill transfer increased from 3.55 to 3.84 and 4.03. The same pattern is visible in Figure 6, which reports the group means for authorship, support, and skill transfer. This is the clearest descriptive evidence that content-generative AI carried a double signal: students reported stronger process support, but the final work displayed weaker authorship evaluation.

**Figure 5:**
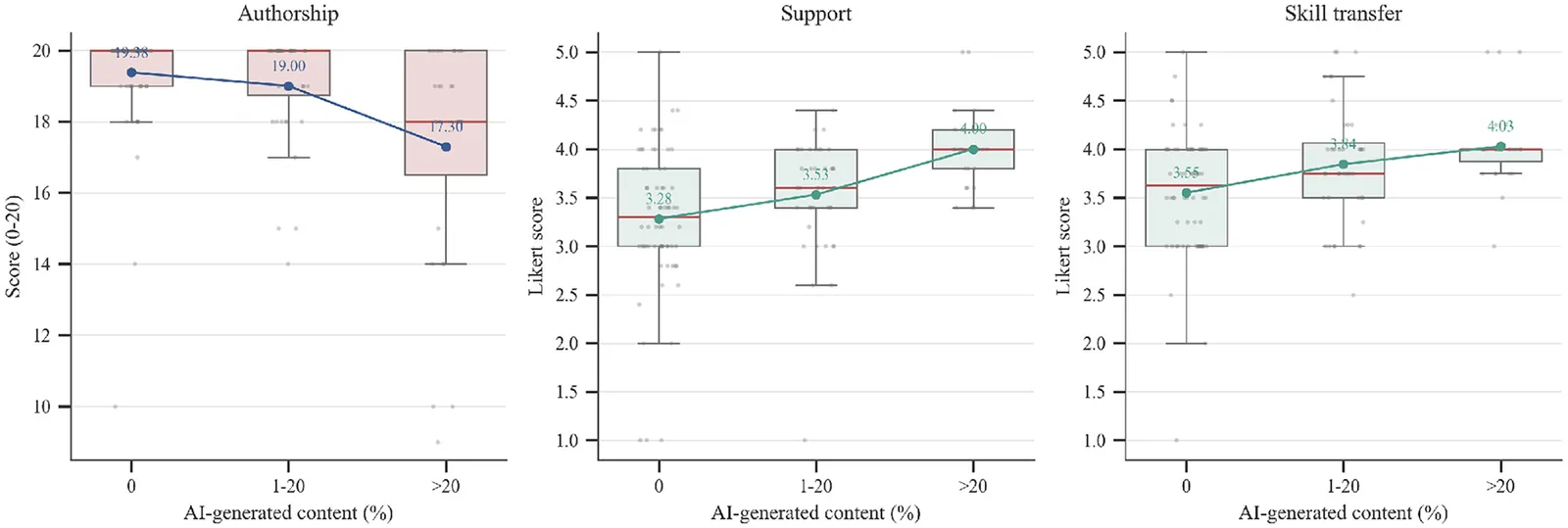
Content-generative AI dose profile for authorship, support, and skill transfer. Group means are overlaid on boxplots.

**Figure 6:**
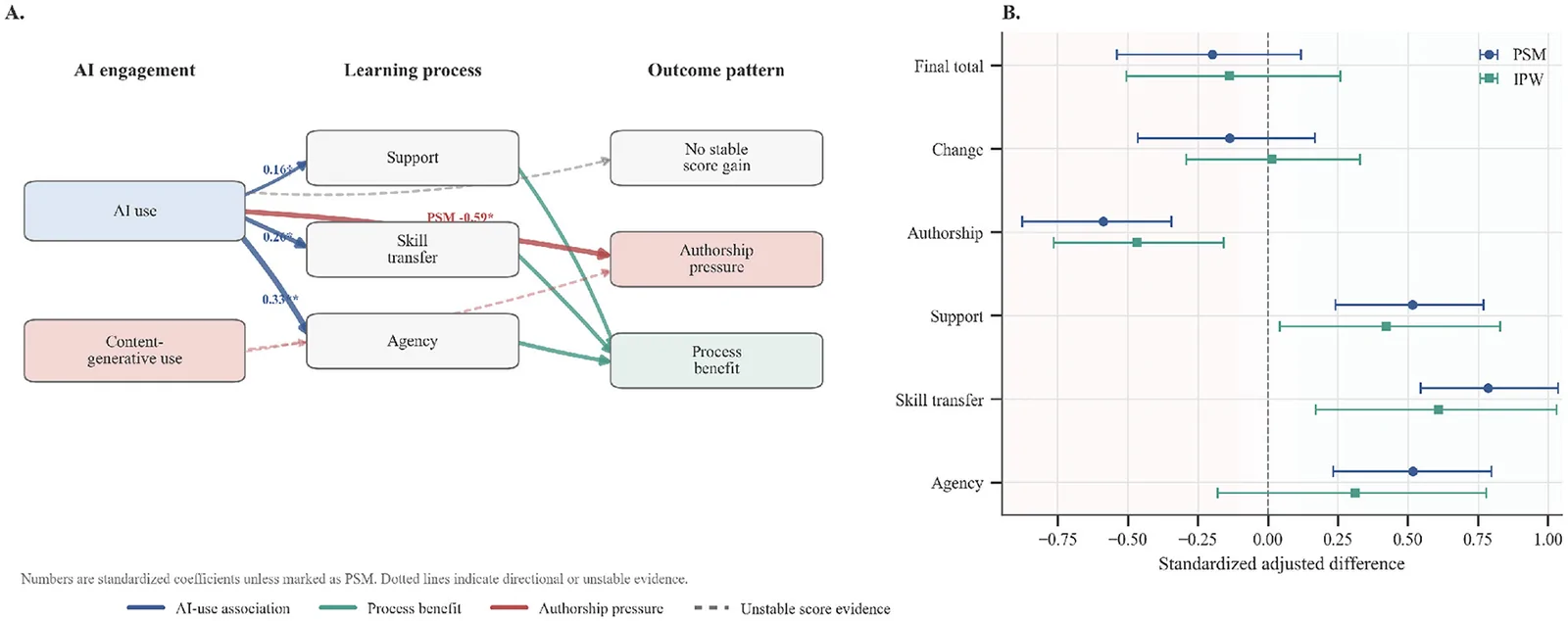
Model-based synthesis of AI-use pathways and propensity-adjusted AI-user contrasts. **(A)** Model-based synthesis. **(B)** Propensity-adjusted contrasts. Numbers are standardized coefficients unless marked as PSM. Dotted lines indicate directional or unstable evidence.

The authorship pattern is not simply a sign that students became passive when AI entered the process. A student may remain active while prompting, selecting, rejecting, revising, and recombining AI outputs. The difficulty is that these decisions may leave little visible trace in the final artifact. Instructors usually evaluate authorship through what the work itself makes visible: composition, style, transformation of references, consistency between intention and form, and evidence of independent decision-making. When AI-generated visual features dominate the submitted work, procedural agency may fail to become visible authorship. This distinction also echoes recent work on human–AI creative agency, where control, ownership, and self-efficacy may fluctuate across the creative process rather than appearing as a single stable condition (Rafner et al., 2025).

This distinction between procedural agency and visible authorship helps explain why content-generative use was associated with a stronger authorship tension than assistive use. Assistive AI can support reference search, ideation, technical clarification, or iterative feedback while leaving the main visual decisions more clearly attached to the student. Content-generative AI enters the visible layer of form-making. Even when the student repeatedly revises prompts and directs the model according to personal intention, the final artifact may still carry an AI-generated appearance. In legal and creative-authorship debates, a similar problem appears in the difficulty of specifying how much human contribution is sufficient for an AI-assisted output to be recognized as meaningfully human-authored (Militsyna, 2023). In that situation, the student's agency exists in the process, but it is less legible in the final work. Authorship, in creative education, is not only exercised; it must also be made visible.

The issue is therefore not whether authorship evaluation is fair or unfair in a simple sense. Authorship remains a necessary criterion because the course is not only assessing completion or visual polish. It is also assessing intention, transformation, judgment, and the student's ability to develop a personally accountable visual language. At the same time, AI-mediated work requires richer process evidence. Prompts, iterations, rejected outputs, manual revisions, and reflective explanations may become part of how authorship is demonstrated. This also aligns with portfolio-based art assessment, where sketches, drafts, reflection, and revision records are treated as evidence of artistic thinking rather than peripheral documentation. Without such evidence, evaluators are left mainly with the final image, and the final image may not show enough of the student's decision-making. This point connects the present findings with learning theories that emphasize active and constructive engagement (Chi, 2009) and with cognitive load theory, which suggests that external systems can support performance while also changing the distribution of internal processing (Sweller et al., 2007).

The pathway checks add context to this interpretation without becoming the main claim. The strongest directional estimates clustered around content-generative use, skill transfer, creative experience, and authorship. The broader pattern is consistent with mediation-oriented analysis, where direction, interval estimates, and model consistency are considered together rather than reduced to a single stepwise test (MacKinnon et al., 2004; Preacher and Hayes, 2008). In the present results, the clearest signal is a process–outcome separation: AI use shows a clearer and more consistent pattern in process experience than in final score gain, and authorship is the dimension where this separation becomes visible.

Taken together, the findings answer H1 and H2 in a differentiated way. H1a is not supported as a broad final-score advantage of AI use. H1b receives limited support in the adjusted performance models, because assistive use did not produce stable final-score gains after baseline performance was considered. H1c receives partial support: the authorship dimension showed the strongest AI-related pattern, especially in the adjusted AI-user contrasts, the content-generative dose profile, and the authorship sensitivity analyses. H2a receives exploratory support at the pattern level, as AI use showed positive unadjusted associations with learning-process variables and consistent positive directions in propensity-adjusted comparisons. H2b is partially supported, because the mode and intensity of AI use mattered, while assistive and content-generative practices partly overlapped. H2c and H2d receive limited support, since learning-process variables did not form a broad route into higher artistic performance. The main conclusion is therefore not that AI directly improves creative outcomes, but that AI is associated with changes in the conditions under which creative work is supported, completed, and recognized as the student's own.

### 4.3 AI use, psychological perception, and the visibility of authorship

The RQ3 analysis examines whether AI use changes students' psychological perceptions of their work and whether these perceptions appear in artistic evaluation. The pattern is not a simple confidence gain. AI use was associated with stronger confidence in the completed artifact and stronger willingness to identify the work as displayable, but these gains did not become broad advantages in final artistic performance. The strongest tension appeared in content-generative AI use: as the reported proportion of AI-generated content increased, students' quality confidence and display identity rose, while externally evaluated authorship declined.

Figure 7 and Table 7 summarize the baseline-adjusted models linking AI use to psychological variables. Binary AI use was positively associated with quality confidence (β_*std*_ = 0.36, *p* < 0.001) and display identity (β_*std*_ = 0.29, *p* < 0.001), with a marginal positive association with display readiness (β_*std*_ = 0.15, *p* < 0.10). By contrast, AI use showed no stable association with internalization, creative confidence, self-evaluation, or gain confidence. Assistive and content-generative intensity, when entered together, did not show stable independent coefficients across most psychological variables. This difference is meaningful. Binary AI use captures entry into an AI-supported workflow, while the intensity measures describe variation in how much support or generated content students reported within that workflow. The results therefore support H3a in a specific sense: AI use was associated with stronger presentation-oriented self-perception, but it did not broadly strengthen deeper confidence, internalization, or self-evaluation.

**Figure 7:**
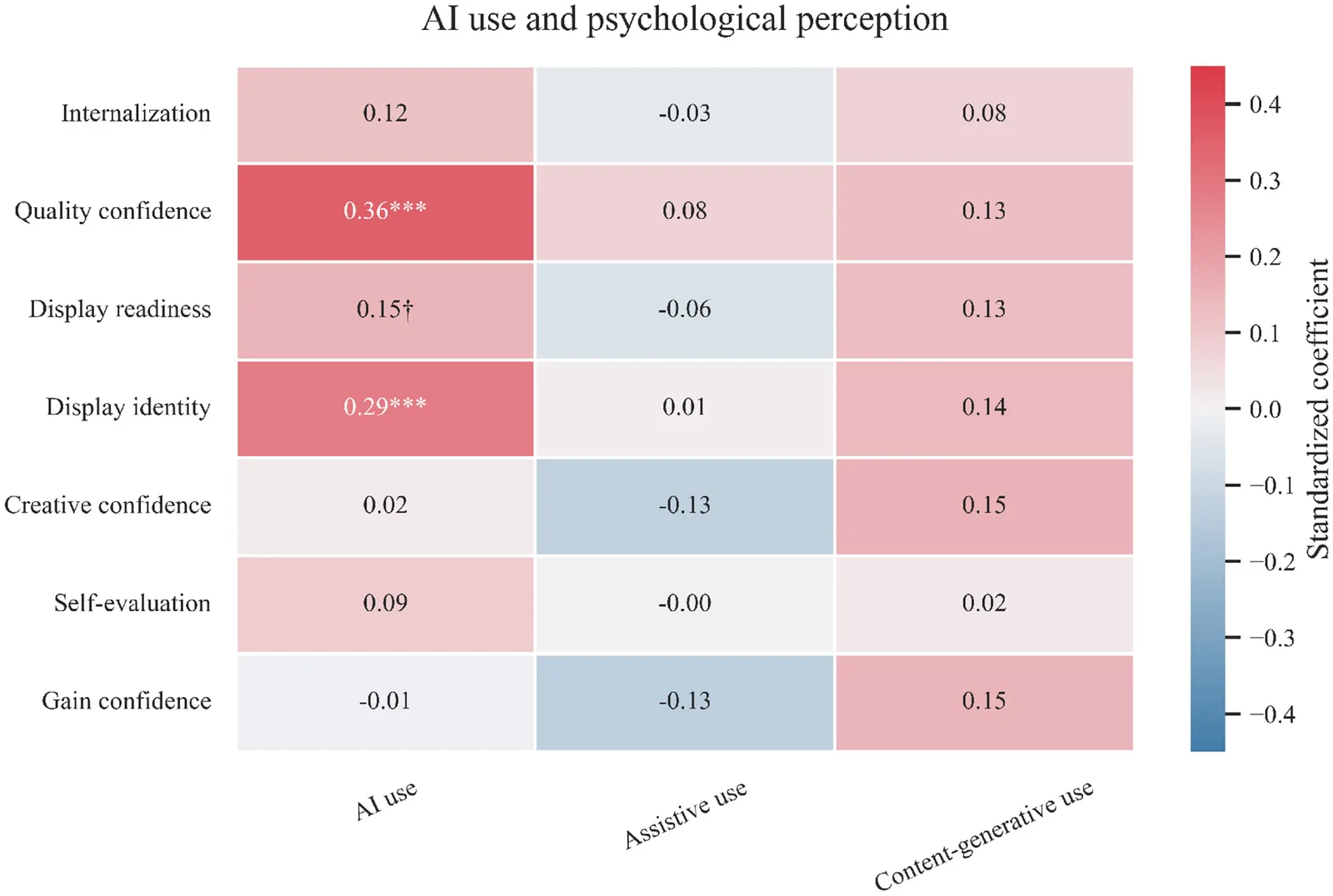
AI use and psychological perception. Standardized coefficients are reported from HC3 robust OLS models controlling for daily score, mid-term score, and prior AI experience. ^†^*p* < 0.10, ^***^p < 0.001.

**Table 7:** AI use and psychological perception.

Outcome	β_AI use_	*p*	β_Assistive_	*p*	β_Content_	*p*
Internalization	0.12	0.234	−0.03	0.757	0.08	0.572
Quality confidence	0.36	<0.001	0.08	0.480	0.13	0.281
Display readiness	0.15	0.083	−0.06	0.644	0.13	0.370
Display identity	0.29	<0.001	0.01	0.938	0.14	0.280
Creative confidence	0.02	0.872	−0.13	0.345	0.15	0.270
Self-evaluation	0.09	0.362	−0.00	0.992	0.02	0.855
Gain confidence	−0.01	0.943	−0.13	0.495	0.15	0.373

Values are standardized coefficients from HC3 robust OLS models. All models control for daily score, mid-term score, and prior AI experience. Assistive and content-generative AI use are entered together in differentiated models. Internalization was computed as the mean of agency, self-evaluation, perceived gain in artistic ability, and perceived process clarity.

Students who used AI were not more confident in every sense. Their confidence was concentrated around the finished work as an object that could be recognized, displayed, and accepted by others. Such confidence is educationally meaningful because public presentation is part of creative practice. At the same time, it differs from internalized artistic growth. A student may feel that a work looks polished and exhibition-ready without feeling that the process has become fully internalized as personal artistic capacity (Zimmerman, 1990). The distinction suggests that AI may strengthen confidence in the artifact rather than confidence in the self as a developing creator (Bandura, 1977; Tierney and Farmer, 2002).

The content-generative dose profile provides the clearest evidence of this separation. As shown in Figure 8 and Table 8, students who reported more than 20% AI-generated content had the lowest authorship scores but the highest levels of quality confidence. Authorship declined from 19.38 in the no-content group to 19.00 in the 1%–20% group and 17.30 in the group above 20%. Display identity increased from 3.26 to 3.64 and 3.62. Quality confidence also increased from 3.36 to 3.85 and 3.84. This profile should be read together with the baseline-adjusted performance models reported in RQ2, where prior performance remained central to final outcomes. Even so, the group pattern is theoretically important: content-generative AI was associated with a visible divergence between students' self-perceived readiness and the evaluated authorship of the final work. These results support H3c.

**Figure 8:**
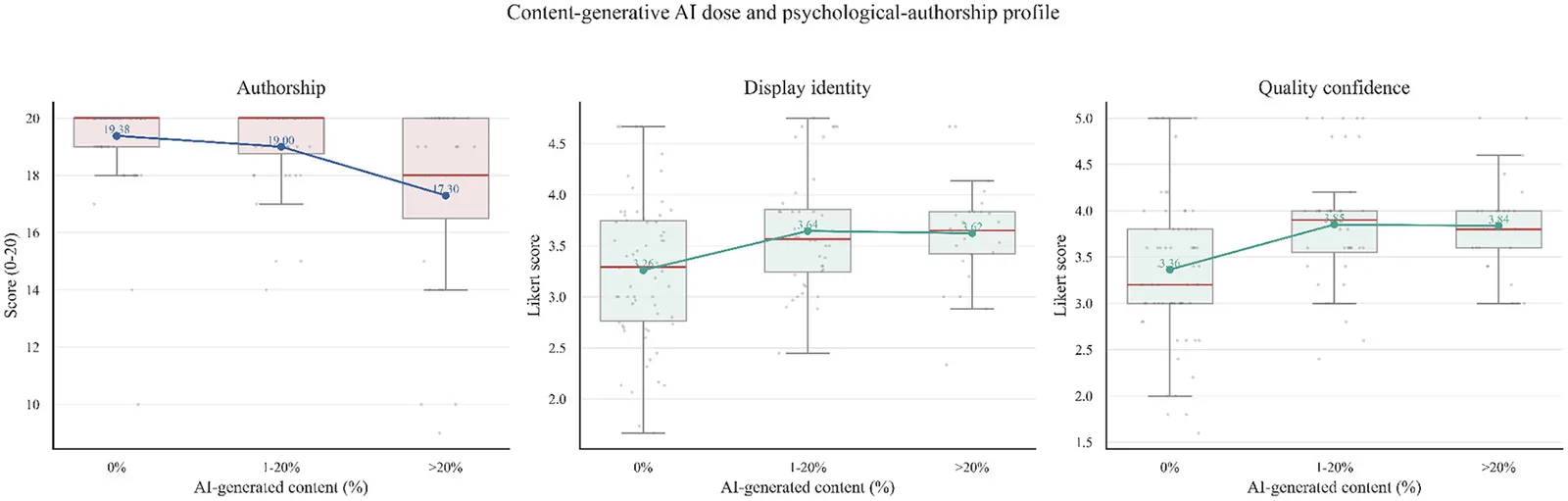
Content-generative AI dose and psychological-authorship profile. Group means are overlaid on boxplots.

**Table 8:** Content-generative AI dose, psychological perception, and authorship.

AI-generated content	*N*	Authorship	Display identity	Quality confidence	Internalization
0%	66	19.38	3.26	3.36	3.93
1%–20%	40	19.00	3.64	3.85	4.00
>20%	27	17.30	3.62	3.84	4.01

Values are group means. Authorship is scored on a 0–20 scale. Display identity, quality confidence, and internalization are measured on 1–5 scales. ANOVA results: authorship *F* = 10.34, *p* < 0.001, η^2^ = 0.137; display identity *F* = 5.85, *p* = 0.004, η^2^ = 0.083; quality confidence *F* = 7.62, *p* < 0.001, η^2^ = 0.105; internalization *F* = 0.33, *p* = 0.722, η^2^ = 0.005.

The direction of this pattern should not be reduced to a simple loss of student agency. Students may remain active while prompting, revising, selecting, rejecting, and recombining AI outputs. What changes is the visibility of that agency in the submitted artifact. Content-generative AI enters the visible layer of form-making. When the final image carries strong AI-generated visual features, the student's decisions may be present in the process but less legible in the work itself (Rafner et al., 2025; Militsyna, 2023). The evaluator sees the finished artifact, not every prompt, rejected output, adjustment, and manual correction behind it. In this sense, content-generative AI can strengthen students' confidence in presentation while weakening the visible evidence of personal authorship.

The models linking psychological variables to artistic performance further clarify this point. As reported in Table 9, psychological variables did not show a broad predictive pattern across final total score, performance change, creativity, aesthetic quality, technique, authorship, or completion after controlling for prior performance and AI experience. Display readiness had only a marginal association with completion (β_*std*_ = 0.17, *p* < 0.10). Other coefficients remained small and inconsistent. These results do not support H3b. Psychological perception did not function as a general route into higher artistic scores.

**Table 9:** Psychological perception and artistic performance.

Outcome	Internal.	Quality conf.	Display ready	Display identity	Creative conf.	Self-eval.	Gain conf.
Final total	0.07	0.04	0.12	0.09	−0.00	0.12	−0.01
Change	0.07	0.04	0.13	0.09	−0.00	0.13	−0.01
Creativity	0.08	0.05	0.09	0.08	−0.00	0.14	−0.01
Aesthetic	0.09	0.11	0.12	0.13	0.03	0.13	0.03
Technique	0.06	0.06	0.12	0.09	0.00	0.10	−0.00
Authorship	−0.01	−0.12	0.03	−0.05	−0.06	0.04	−0.09
Completion	0.10	0.11	0.17^†^	0.15	0.03	0.13	0.04

Values are standardized coefficients from separate HC3 robust OLS models. All models control for daily score, mid-term score, and prior AI experience. ^†^*p* < 0.10.

The pathway tests reported in Table 10 locate where this psychological shift occurred. Assistive AI use showed significant indirect pathways through support and skill transfer: assistive use was linked to quality confidence through perceived support, and to internalization through skill transfer. In contrast, content-generative pathways into authorship were weak or unstable. The pathway from content-generative use through creative experience to authorship was negative and marginal, while the path through display identity did not reach stable significance. These results provide limited support for H3d. AI-related psychological changes occurred mainly inside the process and self-perception layers; they did not reliably transfer into externally evaluated artistic performance.

**Table 10:** Bootstrap pathway checks for psychological mechanisms.

Pathway	Indirect effect	CI low	CI high	*p*
Assistive → support → quality confidence	0.135	0.029	0.278	0.004
Assistive → skill transfer → internalization	0.160	0.079	0.259	<0.001
Content → internalization → final total	0.004	−0.016	0.033	0.756
Content → display identity → authorship	−0.002	−0.053	0.019	0.766
Content → skill transfer → authorship	−0.024	−0.080	0.016	0.242
Content → creative experience → authorship	−0.027	−0.068	0.002	0.072

Indirect effects are based on 5,000 bootstrap resamples. Variables are standardized in pathway models. All pathway models control for daily score, mid-term score, and prior AI experience.

These findings align with prior work showing that AI can support ideation, perceived creativity, and engagement (Li et al., 2026; Alghamdi, 2025). They also refine that literature by showing that psychological confidence and evaluated artistry need to be separated in creative education. In structured domains, AI assistance may more directly improve measurable task outcomes (Alblooshi, 2026). In studio-based creative work, however, evaluation depends on originality, authorship, expressive coherence, and visible transformation of ideas. AI-generated content can support production while also making personal creative contribution harder to read (Kasneci et al., 2023). This result is consistent with cognitive offloading theory: external systems may reduce effort and support task progress, while also changing what learners internalize and what evaluators can identify as student-owned work (Gerlich, 2025).

From an educational perspective, the evidence suggests that AI changes the psychological surface of creative learning before it changes artistic evaluation. Students may feel more prepared to present and defend a polished work, yet the same work may provide weaker evidence of independent authorship. This is not a minor assessment problem. In creative education, confidence, display readiness, and public identity matter, but they do not replace the need for visible artistic decision-making. AI-mediated assignments therefore require stronger process evidence, such as prompt histories, iteration records, rejected outputs, manual revisions, and reflective explanations (Rafner et al., 2025). These materials can help make student agency visible rather than leaving authorship to be inferred only from the final image (Zawacki-Richter et al., 2019).

Overall, RQ3 suggests that AI use was more clearly associated with psychological perception than with evaluated artistic performance. H3a is partially supported because AI use was associated with quality confidence and display identity, but not with a broad strengthening of internalization or creative confidence. H3b is not supported because psychological variables did not consistently predict artistic performance. H3c is supported at the descriptive pattern level by the content-generative dose profile, where higher reported AI-generated content coincided with higher display identity and quality confidence but lower evaluated authorship. H3d receives limited support: pathway evidence appeared within process and psychological variables, but did not extend into final artistic scores. The central finding is therefore a psychological–authorship split: AI use was associated with stronger presentation-oriented confidence, while content-generative use was associated with less visible student authorship in the evaluated work.

### 4.4 AI use, internalization, teaching climate, and development-oriented outcomes

The final part of the analysis examines whether AI use is connected with students' longer-term creative development. The outcomes considered here include continuation intention, institutional collaboration, social value orientation, and an overall development orientation index. These variables move beyond immediate artistic performance and ask whether students see their creative work as something worth continuing, sharing, developing, and connecting with broader institutional or social contexts. The results show a clear pattern: AI use itself is not the main predictor of these development-oriented outcomes. Instead, the strongest signals come from students' internalization of the creative process and from the perceived AI-related teaching climate.

Table 11 summarizes the key full-model results. After controlling for prior course performance and AI experience, binary AI use remains close to zero across all development-oriented outcomes. Its standardized coefficients range from 0.04 to 0.07 and none reaches statistical significance. The same pattern appears for differentiated AI intensity: neither assistive use nor content-generative use showed a stable independent association in the full models. By contrast, internalization is consistently positive. It is strongly associated with continuation intention (β = 0.51, *p* < 0.001), institutional collaboration (β = 0.28, *p* < 0.05), social value orientation (β = 0.28, *p* < 0.05), and overall development orientation (β = 0.40, *p* < 0.001). Teaching AI climate also shows stable positive associations with institutional collaboration, social value orientation, and development orientation, as visualized in Figure 9.

**Table 11:** Full models for development-oriented outcomes.

Outcome	AI use	Internalization	Teaching AI climate	*R* ^2^
Continuation intention	0.04	0.51^***^	0.18	0.389
Institutional collaboration	0.07	0.28^*^	0.27^*^	0.275
Social value orientation	0.06	0.28^*^	0.33^*^	0.355
Development orientation	0.06	0.40^***^	0.29^*^	0.387

Values are standardized coefficients from full models. All models include AI-use intensity variables, daily score, mid-term score, and prior AI experience as controls. ^*^p < 0.05, ^***^p < 0.001.

**Figure 9:**
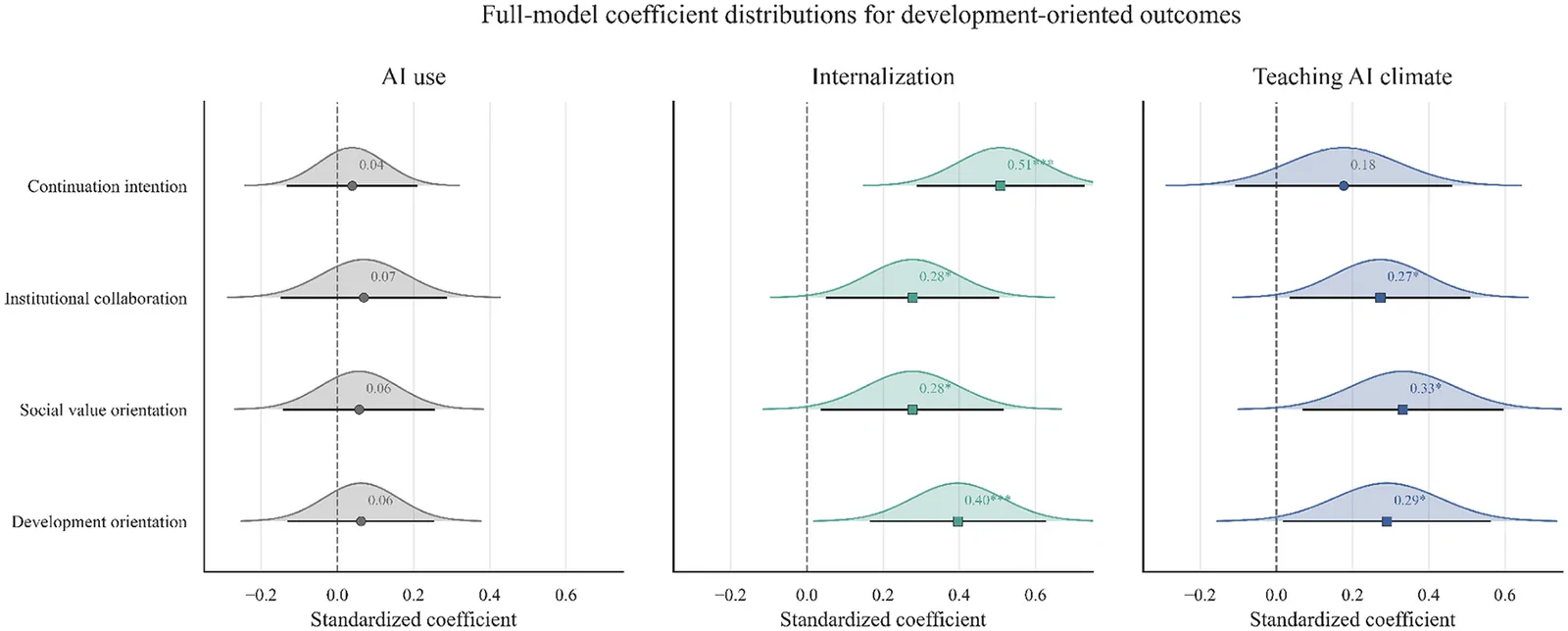
Full-model coefficient distributions for development-oriented outcomes. Points indicate standardized estimates, horizontal lines indicate 95% confidence intervals, and ridge shapes indicate coefficient uncertainty.

These results answer H4 in a differentiated way. H4a is not supported: assistive AI use does not directly predict development-oriented outcomes once prior performance and other model terms are included. H4b is supported: content-generative AI use was not stably associated with positive developmental outcomes across model specifications. H4c is strongly supported: internalization is the central predictor of development-oriented outcomes. The evidence therefore points away from a usage-volume explanation and toward a learning-internalization explanation. Students' future-oriented creative development is shaped less by whether they used AI than by whether the creative process became part of their own reflective and developmental trajectory.

Figure 10 makes this pattern visible at the descriptive level. Across all four outcomes, students in the high-internalization group report stronger development-oriented responses than those in the low-internalization group. Continuation intention rises from 3.28 to 4.28, institutional collaboration from 3.23 to 3.89, social value orientation from 3.36 to 3.93, and development orientation from 3.29 to 4.03. This gradient is important because it shows that developmental orientation is not merely a general attitude toward AI. It is tied to whether students experienced the project as something they understood, owned, and could carry forward. This interpretation is consistent with internalization theory, where sustained engagement depends on whether external learning activities are integrated into learners' own goals and sense of agency (Deci and Ryan, 2000).

**Figure 10:**
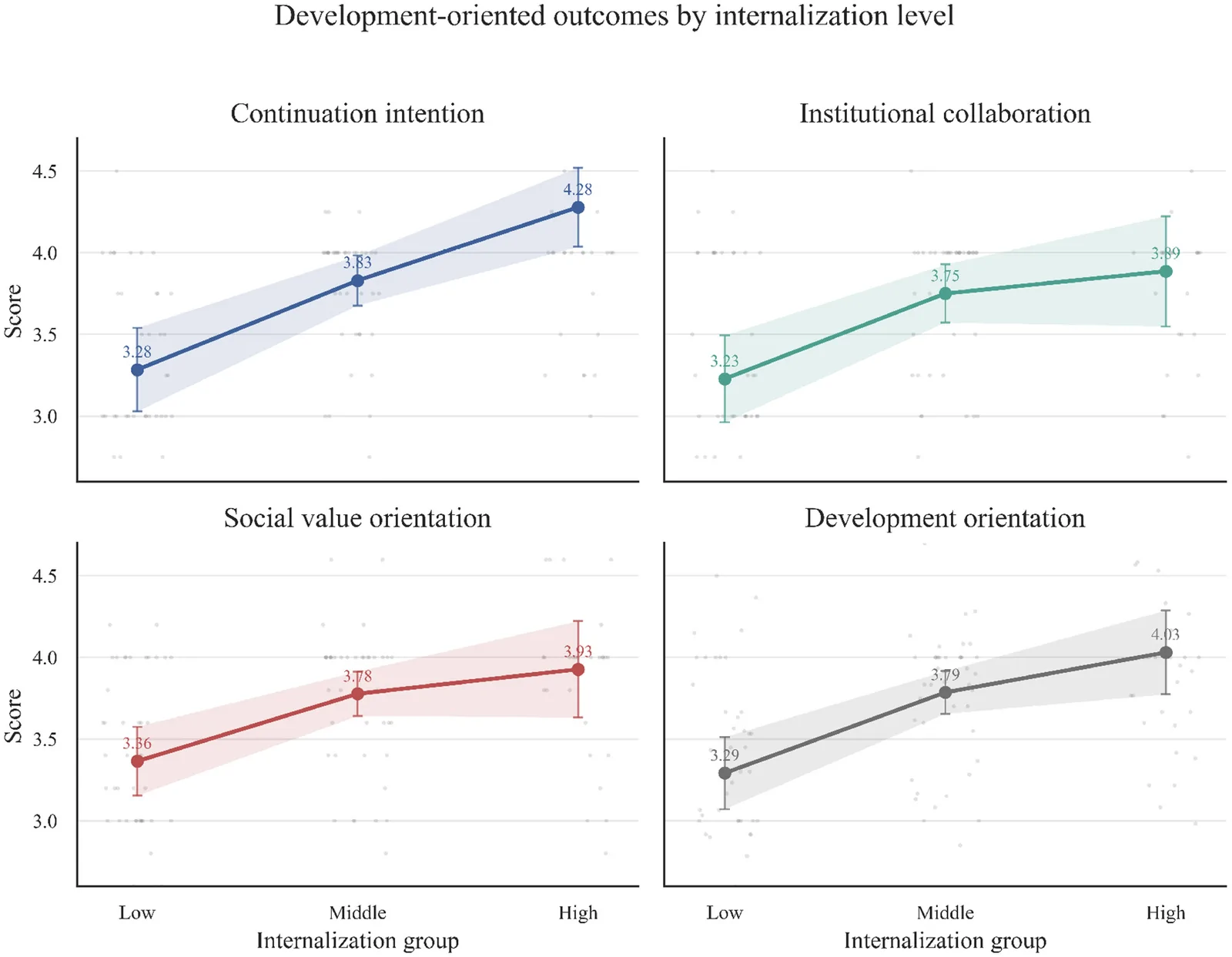
Development-oriented outcomes by internalization level. Students were divided into low, middle, and high internalization groups. Points show group means, shaded areas indicate confidence intervals, and gray dots show individual observations.

The AI-users-only analysis further clarifies this point. Within the subgroup of AI users, AI-help percentage and AI-generated-content percentage remain weak predictors of developmental outcomes. Teaching AI climate, however, becomes especially relevant for institutional collaboration, social value orientation, and development orientation. This means that among students who already used AI, the decisive difference is not simply using more AI or generating more content through AI. The stronger difference lies in whether the course environment helped students make sense of AI use as part of a broader creative, collaborative, and developmental process. This pattern also echoes situated learning perspectives, in which learning becomes durable when students move from task completion toward participation in a wider community of practice (Lave and Wenger, 1991).

This finding also extends the earlier results on psychological perception and authorship. In RQ3, AI use was associated with stronger artifact-facing confidence, but those psychological variables did not translate directly into higher artistic scores. The present results show where psychological engagement does matter: it matters for development-oriented learning. Creative confidence, process ownership, and internalized understanding do not necessarily raise a final score in a tightly evaluated artistic task, but they do shape whether students imagine continuing the work, collaborating with institutions, and assigning social value to their creative outcomes. This distinction is consistent with prior work on AI-supported engagement and metacognitive learning (Zawacki-Richter et al., 2019), deep approaches to learning (Entwistle and Peterson, 2004), and self-efficacy and self-regulated learning perspectives, where sustained learning depends on learners' perceived control, reflection, and capacity to continue acting in the domain (Bandura, 1977; Zimmerman, 1990).

Theoretically, these results reposition AI in creative education. Unlike structured learning environments where AI assistance may directly improve measurable performance (Risko and Gilbert, 2016), AI is not functioning here as a direct engine of long-term development. It operates more indirectly, through the learning conditions that surround its use and through the degree to which students internalize the creative process. This interpretation aligns with sustainable learning perspectives that emphasize internal motivation, reflection, and learner agency as foundations for long-term educational impact (Baidoo-Anu and Ansah, 2023). This is especially important in creative domains, where learning value cannot be reduced to efficiency or output quality alone. A technically polished AI-supported artifact may still have limited educational value if students do not understand how the work developed, how their own choices shaped it, or how it can be extended beyond the assignment. Conversely, when students internalize the process, the project becomes a platform for continuation, collaboration, and social meaning.

Overall, RQ4 suggests that development-oriented outcomes were more consistently associated with internalization and teaching climate than with AI use itself. This finding complements the earlier performance and psychological analyses. AI use may change the learning environment, but its longer-term educational value depends on whether students can convert that experience into internalized creative understanding. For creative education, the practical implication is direct: AI integration should be designed less around tool adoption and more around reflection, authorship, process explanation, and pedagogical support.

## 5 Implications for AI-supported creative learning in higher education art contexts

The findings of this study point to a central conclusion: in higher education art contexts, the educational value of generative AI appears to be shaped less by whether students use AI than by how AI enters the creative process. Across the empirical analyses, AI use did not operate as a direct and stable route to higher artistic performance. Baseline artistic performance remained the strongest predictor of final outcomes, while the direct coefficients of AI use were weak or inconsistent. This pattern does not weaken the role of AI in creative education; rather, it clarifies where that role may be located. AI use was associated with several conditions of creative learning, including support, skill transfer, confidence, display readiness, authorship visibility, internalization, and classroom climate. Its clearest empirical pattern therefore appeared in the organization of learning experience rather than in immediate score production (Zawacki-Richter et al., 2019; Lim et al., 2023).

This result requires a shift in how AI-supported creative education is evaluated. A usage-centered view tends to ask whether AI improves or harms performance. The evidence here suggests a more precise question: what kind of AI use supports which part of the learning process, and at what cost to authorship, agency, and internalized competence? Assistive AI use is more closely connected with process-level gains, especially perceived support and skill transfer. This form of use allows students to obtain feedback, expand references, test alternatives, and refine ideas without necessarily replacing their own creative decisions. In contrast, content-generative AI use shows a different profile. It may improve students' sense of readiness or efficiency, but it also creates pressure around authorship visibility. The problem is not simply that students stop making decisions. In many cases, students may continue to revise, select, prompt, compare, and direct the AI output. Yet when the final artifact carries a strong AI-generated appearance, the student's creative intention becomes harder for evaluators to identify. Authorship is therefore not only a matter of internal agency; it is also a matter of visible artistic trace. This distinction is especially important in art education, where the assessed object must communicate both technical quality and the learner's own creative position (Lee et al., 2024; Chi, 2009; Risko and Gilbert, 2016).

The study also shows a process–outcome separation. AI-supported learning can strengthen engagement with the task while leaving artistic scores largely unchanged. This is not a contradiction. In creative education, process gains and performance gains follow different temporal rhythms. Support, confidence, and skill transfer may emerge during the project, whereas evaluated artistic quality depends on prior ability, visual judgment, technical control, and the coherence of the final work. The limited indirect effects observed in the pathway analyses therefore suggest that AI-supported process gains require pedagogical consolidation before they can become stable creative competence. Without such consolidation, AI may increase activity, fluency, and production speed while leaving deeper artistic development uneven. This interpretation aligns with research on cognitive engagement and cognitive offloading, which warns that external support can be productive only when learners remain actively involved in planning, judgment, and reflection (Chi, 2009; Risko and Gilbert, 2016).

The psychological results extend this argument. AI use is associated with several psychological perceptions, but these perceptions are only weakly connected with evaluated artistic outcomes. Students may feel more prepared to present, more confident about quality, or more aware of the display value of their work, while the final assessment still depends on whether the work demonstrates artistic authorship, technique, and conceptual originality. This gap matters because creative education cannot rely on confidence alone. Confidence becomes educationally meaningful when it is connected to internalization: the learner's ability to translate external support into personal judgment, transferable skill, and future creative direction. In the final models, internalization and teaching AI climate are more consistently related to development-oriented outcomes than AI use itself. This indicates that longer-term learning depends less on tool adoption and more on whether students can make AI-supported work their own within a clear pedagogical environment (Chen et al., 2020).

The open-ended responses provide further evidence for this interpretation. Figure 11 shows that students' descriptions are organized around four major thematic groups: support and efficiency, learning and development, authorship and dependence, and fairness and evaluation. The largest cluster concerns support and efficiency, accounting for 41.4% of coded keyword mentions, with frequent references to assistance, inspiration, efficiency, and iteration. This confirms that students experience AI primarily as a process-support tool. At the same time, authorship and dependence form another large cluster, accounting for 32.8% of coded mentions. The co-occurrence network shows that terms related to assistance, inspiration, authorship, control, and dependence are not isolated. They appear together in students' reflections, suggesting that the perceived benefit of AI is accompanied by a visible concern about creative ownership. Learning and development form a third cluster, while fairness and evaluation remain smaller but conceptually important. These qualitative patterns reinforce the quantitative findings: AI-supported creative learning is not a simple improvement story, but a mixed mechanism in which support, dependence, authorship, and evaluation are closely entangled (Sabbaghan and Eaton, 2025).

**Figure 11:**
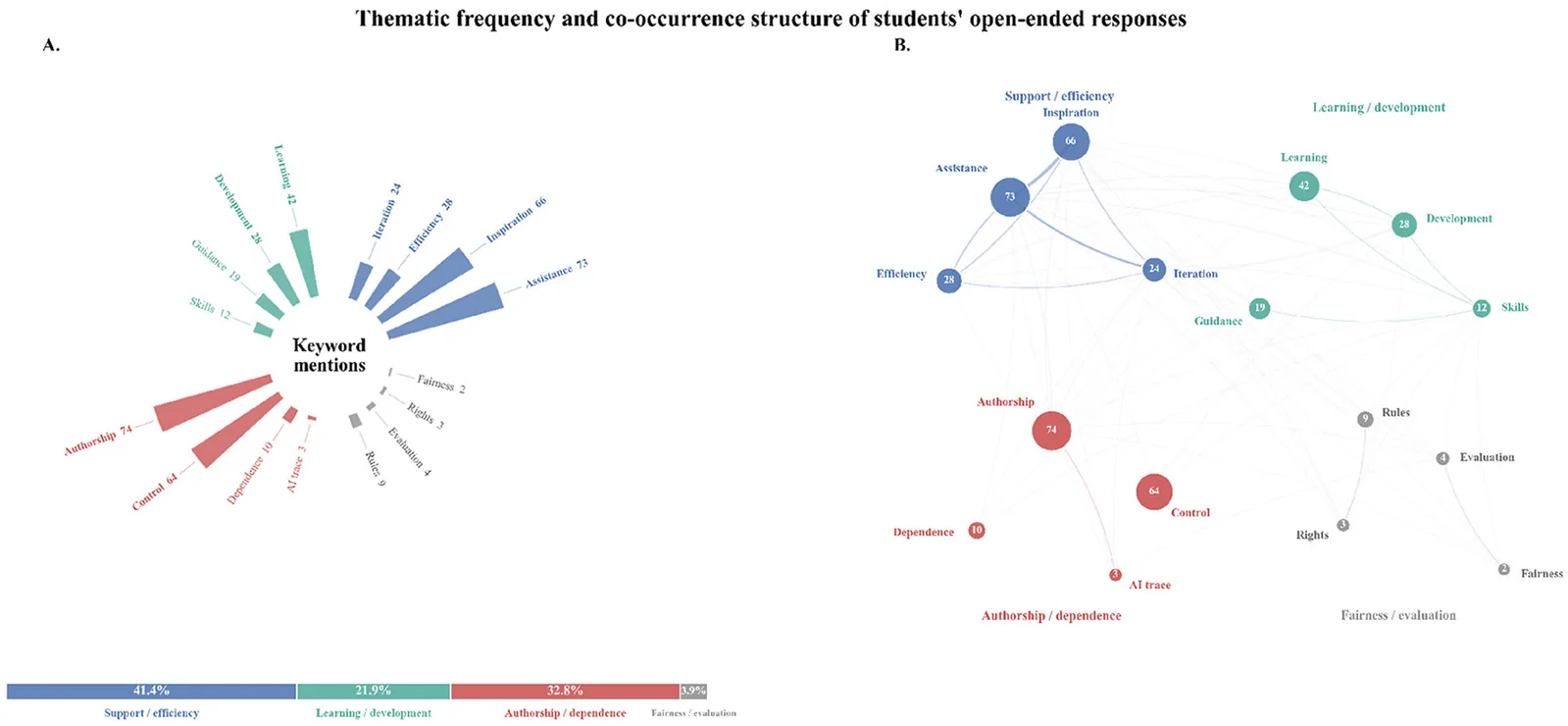
Thematic frequency and co-occurrence structure of students' open-ended responses on AI-supported creative learning. **(A)** Keyword mentions with scale-adjusted radial display. **(B)** Keyword co-occurrence network. Counts are based on keyword-assisted coding of open-ended responses (*N* = 133). Percentages indicate the share of coded keyword mentions by theme. Node size shows keyword frequency; line width shows co-occurrence in the same student's response. Themes are descriptive and not mutually exclusive.

The practical implication is that AI integration in art education should move from access provision to learning design. Students need opportunities to use AI as a partner for exploration, critique, and refinement, but they also need explicit requirements for documenting decisions, explaining prompts, showing iterations, and identifying which parts of the work reflect their own judgment. Such practices make authorship visible without reducing creativity to a mechanical disclosure checklist. They also help teachers distinguish between productive AI-supported authorship and superficial AI substitution. In this sense, responsible AI-supported creative learning depends on three pedagogical conditions: preserving the learner's decision-making role, strengthening internalization through reflection and revision, and maintaining a classroom climate in which AI use is discussed through criteria of quality, authorship, and fairness rather than treated only as a technical convenience.

Overall, this study suggests that generative AI should be understood as a mechanism-dependent component of creative education. Its value is not located in automatic performance improvement, but in how it reorganizes the relationship between support, agency, authorship, confidence, and future-oriented development. For higher education art courses, the central task is therefore not to maximize AI use, nor to prohibit it, but to design conditions under which AI-supported activity becomes visible creative learning. When AI use is connected to reflection, authorship articulation, and internalization, it can contribute to sustained creative development. When it remains a shortcut to output, it risks weakening the very forms of judgment and ownership that art education is meant to cultivate.

## 6 Conclusion

This study examined how generative AI becomes part of creative learning in higher education art and design courses. The central finding is not that AI use directly improves or harms artistic performance, but that different forms of AI use are associated with different layers of creative learning. After accounting for prior course performance, AI use was not stably associated with higher final total scores or baseline-to-final performance change. This means that AI should not be understood as a simple performance booster in this course-based dataset. Instead, its educational relevance appeared most clearly in the relation between process support, visible authorship, psychological perception, and students' internalization of the creative process.

For artistic performance, the strongest predictor of final outcomes was students' earlier course performance rather than AI use itself. Binary AI use, assistive AI use, and content-generative AI use did not show a stable broad advantage across final total score, creativity, aesthetic quality, technique, completion, or performance change after baseline performance was considered. The clearest AI-related performance pattern appeared in authorship. Students with greater AI involvement, especially visible content-generative involvement, tended to receive lower evaluated authorship scores. This pattern was consistent across propensity-adjusted comparisons and alternative sensitivity analyses for ceiling-concentrated authorship outcomes. However, it should be interpreted as an association rather than as evidence that AI directly reduced authorship. The finding points to a key assessment issue: students may remain active in prompting, selecting, revising, and recombining AI outputs, but these decisions may not be visible enough in the final artifact to be recognized as student authorship.

For learning-process outcomes, AI use was more clearly associated with students' experience of the creative process than with externally evaluated artistic performance. AI users reported stronger perceived support, skill transfer, aesthetic judgment, and agency in the unadjusted models, although these associations should be interpreted cautiously because they did not remain significant after FDR correction. The propensity-adjusted comparisons showed a similar process-oriented pattern, especially for perceived support and skill transfer. At the same time, the separate AI-help and AI-generated-content intensity measures did not show stable independent associations when entered together, suggesting that assistive and content-generative practices partly overlapped in students' actual workflows. Overall, the learning-process findings suggest that AI may help students experience creative work as more manageable, exploratory, and transferable, but this process-level support did not translate into a broad final-score advantage.

The psychological findings further clarify this process–outcome separation. AI use was associated with stronger quality confidence and display identity, but not with a broad strengthening of internalization, creative confidence, self-evaluation, or evaluated artistic performance. The content-generative dose profile showed a particularly important split: students reporting higher AI-generated content tended to show stronger quality confidence and display identity, while also receiving lower evaluated authorship scores. This suggests that AI-supported work may feel more polished or presentation-ready to students while still raising questions about how much independent creative intention is visible in the final artifact. Psychological confidence, therefore, should not be treated as equivalent to assessed artistic development or visible authorship.

For development-oriented outcomes, AI use itself was not the most consistent predictor. Continuation intention, institutional collaboration, social value orientation, and overall future development were more consistently associated with students' internalization of the creative process and with the perceived AI-related teaching climate. This finding shifts the educational focus from tool adoption to learning design. The longer-term value of AI-supported creative education depends on whether students can turn AI-supported activity into personal understanding, transferable judgment, and a clearer sense of ownership. AI becomes educationally meaningful when it helps students explain their choices, compare alternatives, revise outputs, and connect technical assistance with their own artistic intentions.

Taken together, the study contributes a differentiated account of AI in creative education. It shows that AI-supported creative learning cannot be evaluated only by final output quality or by whether students used AI. The central issue is how AI participates in the creative process, and whether that participation strengthens or obscures student authorship, judgment, and internalization. Responsible AI integration in art education therefore requires assessment designs that make creative decision-making visible, including sketches, prompts, iterations, rejected outputs, manual revisions, and reflective explanations. Under these conditions, AI can be treated not merely as a shortcut to output, but as a context in which students practice authorship, judgment, and creative responsibility.

## 7 Limitations and future work

This study also points to several directions for future research. First, the analysis was conducted in a real course setting at a single institution, using a course-based sample rather than an experimentally assigned design. This setting allowed the study to examine AI-supported creative learning as it occurred in an authentic instructional context, but it also means that the findings should be interpreted as associations rather than as causal effects. Future studies could extend this work across multiple institutions, courses, and art or design programs, and could compare different instructional models for integrating AI into creative education. Such designs would help examine whether the process–outcome pattern observed in this study appears across broader educational contexts.

Second, future research could further refine the measurement of creative development over time. In this study, the baseline score and final score were related course-performance measures rather than identical pre-test and post-test instruments. The performance-change score therefore served as a course-based indicator of movement from earlier performance to final assessment. Future studies could build on this approach by using matched creative tasks, repeated project cycles, or longitudinal portfolios to trace how students' artistic judgment, technical control, authorship awareness, and AI-use habits develop across time.

Third, future work could collect richer process data on how students use AI during creative production. The present study measured AI-help percentage and AI-generated-content percentage through students' self-estimates, which captured their perceived level of AI involvement in the project. To complement such self-report data, future studies could incorporate prompt histories, version records, manual revision traces, screen recordings, process journals, and artifact-level analysis of AI-shaped visual features. These data would make it possible to examine not only whether AI was used, but how students negotiated intention, revision, selection, rejection, and ownership during production.

Fourth, future studies should further investigate how authorship is evaluated in AI-supported creative work. In this study, evaluators did not have access to students' questionnaire responses or reported AI-use status, but the final artifact itself may still have carried visual features associated with AI-shaped production. Future research could compare artifact-only assessment with assessment that includes process documentation, reflective explanation, or prompt-and-revision records. This would help clarify how evaluators interpret visible authorship, how students' prior artistic ability and artwork type interact with AI use, and how assessment criteria can better capture both artifact quality and creative decision-making.

Overall, future research should move toward evaluation frameworks that combine artifact quality, process evidence, and students' own accounts of creative control. This is especially important for art and design education, where learning is not limited to producing a polished image, but includes developing judgment, taste, persistence, and a personal creative position.

## Data Availability

The datasets presented in this article are not readily available because the data used in this study are confidential due to privacy and ethical considerations. However, anonymized datasets may be made available from the corresponding author upon reasonable request, subject to a formal confidentiality agreement.

## Supplementary material

Supplementary material S1 Questionnaire Instrument.
