# Supplementary material for: Generative AI in creative education: baseline-linked evidence on learning processes, artistic performance, and authorship
Source: Front Psychol. 2026 Oct 2;17:1956939. doi: 10.3389/fpsyg.2026.1956939 (PMC13672588; doi:10.3389/fpsyg.2026.1956939)
Supplement: Supplementary file 1 [file Data_Sheet_1.pdf]

AI 工具 与 艺术创作学习问卷

AI Tools and Artistic Creation Learning Questionnaire

本问卷仅用于教学与研究分析，任课教师对 AI 工具辅助艺术创作持绝对开放态度，因此除作品质量外问卷内容不影响课程成绩。
This questionnaire is used solely for teaching and research purposes. The instructor maintains a fully open attitude toward AI-assisted artistic creation; therefore, responses will not affect course grades except for the quality of the submitted work.

任课教师认可 AI 在艺术创作中的多种可能作用，课程评价以作品呈现为主，与是否使用 AI 无直接关系。
所有回答将匿名处理，请根据你本次期末创作作业的真实情况作答。
没有“正确答案”，请根据实际感受放心填写。
The instructor acknowledges the multiple potential roles of AI in artistic creation. Course evaluation is primarily based on the final work, and is not directly related to whether AI is used.
All responses will be processed anonymously. Please answer based on your actual experience in this final project.
There are no “correct answers.” Please respond honestly based on your true experience.

一、基本信息（Basic Information）

1. **你的专业 / 学习方向是（What is your major / field of study?）：**
   ☐ 动画（Animation）
   ☐ 动画（漫插画）（Animation (Illustration / Comics)）
2. **你目前的学习阶段是（What is your current academic level?）：**
   ☐ 本科一年级（Year 1 Undergraduate）
   ☐ 本科二年级（Year 2 Undergraduate）
   ☐ 本科三年级（Year 3 Undergraduate）
   ☐ 本科四年级（Year 4 Undergraduate）
3. **您的性别（Your gender）：**
   ☐ 男（Male）
   ☐ 女（Female）
   ☐ 我不愿意透露（Prefer not to disclose）
4. **你本次提交的作品主要属于以下哪一类（Which category does your submitted work mainly belong to?）？**
   ☐ 动漫角色设计/IP 形象设计（Character Design / IP Design）
   ☐ 动画概念设计（Animation Concept Design）
5. **在本课程之前，你使用 AI 工具进行创作的经验程度是：**
   （Prior to this course, how much experience did you have using AI tools for creative work?）

（1 = 完全没有，5 = 非常丰富）（**1 = None, 5 = Very extensive**）

1 ☐ 2 ☐ 3 ☐ 4 ☐ 5 ☐

6. **在本科阶段学习后您的职业规划属于？（单选）（What are your career plans after completing your undergraduate studies? (Single choice)）**

☐ 升学深造（Further study） ☐ 就业（Employment）

☐ 尚不确定（Not sure） ☐ 其他（Other）：____

（**若选择“升学深造”**）你的升学计划是（单选）（If “Further study” is selected, your plan is）：

☐ 同等级或低于本校等级的高等教育学校学习（Same or lower-ranked institutions）

☐ 更高等级的高等学校学习（Higher-ranked institutions）

☐ 海外高等教育学校学习（Overseas institutions）

（**若选择“就业”**）你更倾向的就业去向是（单选）（If “Employment” is selected, your preferred career path is）：

☐ 自由创作者或自由职业（Freelance）

☐ 政府、事业单位部分的职位（Public sector）

☐ 动漫、影视、文创、传媒类企业（Cultural / Media industries）

☐ 其他（Other）：

7. **你本次期末创作作业的完成方式主要是（How was your final project mainly completed?）：**

☐ 个人独立完成（Individually）

☐ 多人讨论合作完成（Collaborative work）

二、AI 工具的使用情况（AI Tool Usage）

1. **在本次期末创作中，你是否使用了任何 AI 工具？（Did you use any AI tools in this final project?）**

   ☐ 是（Yes）

   ☐ 否（No）（若选择“否”，仍请继续完成后续问题，根据你的实际创作体验作答）

2. **您使用的 AI 工具为（Which AI tools did you use?）**

（请填写（Please specify））

3. **你在本次创作中使用过哪些类型 AI 工具？（可多选）（Which types of AI tools did you use? (Multiple choice)）**

   ☐ 文生图（Text-to-image）

   ☐ 图生图 / 局部修改 / 风格迁移（Image generation / Local modifications / Style transfer）

   ☐ AI 修图 / 增强 / 自动编辑（AI photo editing / Enhancement / Automatic editing）

   ☐ AI 文本生成（概念、说明、叙事等）（AI text generation (concepts, descriptions, narratives, etc.)）

   ☐ AI 音乐 / 声音生成（AI music / Sound generation）

- ☐ AI 视频生成 / 剪辑辅助（AI video generation / Editing assistance）
- ☐ 其他 AI 工具（请注明）（Other AI tools (please specify)）：________
- ☐ 我没有使用任何 AI 工具（I did not use any AI tools）

4. **你在创作的哪些阶段使用了 AI？（可多选）（At which stages of your creative process do you use AI? (Multiple choice)）**

- ☐ 选题 / 灵感阶段（Topic selection / Inspiration phase）
- ☐ 草图 / 初稿阶段（Sketching / First draft phase）
- ☐ 风格探索阶段. （Style exploration phase）
- ☐ 学习技法或操作步骤（Learning techniques or procedures）
- ☐ 生成素材（图像 / 音频 / 文本等）（Creating content (images, audio, text, etc.)）
- ☐ 修改与迭代阶段（Revision and iteration phase）
- ☐ 最终润色阶段（Final polishing phase）
- ☐ 撰写作品说明 / 概念阐释（Writing a description of the work / conceptual explanation）
- ☐ 我未在任何阶段使用 AI（I did not use AI at any stage）

三、AI 的使用方式（Modes of AI Usage）

1. **以下哪一项最接近你在本次创作中使用 AI 的方式？（可多选，如选择多项，请在本题末尾填写你认为各方式的大致比例。总和为 100%，不要求精确。）（Which of the following best describes how you used AI in this project? (ou may select more than one option. If you do, please indicate the approximate percentage for each method at the end of this question. The total should add up to 100%; exact figures are not required.)）**

- ☐ 我主要用 AI 来获取灵感或想法（I mainly used AI to generate ideas or inspiration）
- ☐ 我主要用 AI 来辅助创作过程（反复修改、优化）（I mainly used AI to assist the creative process (iteration and refinement)）
- ☐ 我主要用 AI 来学习技法或操作方法 （I mainly used AI to learn techniques or workflows）
- ☐ 我主要用 AI 生成参考素材/对照样例（用于比对、取舍，而非直接采用）（I mainly used AI to generate reference materials (for comparison, not direct use)）
- ☐ AI 直接生成了作品中较为重要的部分（AI directly generated important parts of the final work）
- ☐ 以上几种方式都有，且比例比较接近（I used multiple approaches in combination）
- ☐ 我没有使用 AI（I did not use AI）

各方式比例（总和≈100%）（Proportion of each method (total ≈100%)）：获取灵感或想法（Inspiration）__% / 辅助创作过程（Process support）__% / 学习技法或操作方法（Learning

techniques）__% / 生成参考素材/对照样例（Reference materials）__% / 重要部分生成（Core generation）__%

2. **AI 对我“推进创作流程”的帮助比例大约是（Approximately what percentage did AI contribute to advancing your creative workflow?）：**
（0–100%，主观判断，不要求精确）（0–100%, subjective assessment）

_____ %

说明：这里的“推进创作流程”指帮助你更快推进进度、减少卡顿、优化步骤、提高效率等。（Note: “streamlining the creative process” refers to helping you make faster progress, reduce bottlenecks, optimize steps, and improve efficiency.）

3. **在最终提交的作品中，AI 生成或直接修改的内容占比大约是（In your final work, what proportion of the content was generated or modified by AI?）：**
（0–100%，主观判断，不要求精确）（0–100%, subjective assessment）

_____ %

说明：这里的“内容占比”指最终作品中由 AI 直接生成、重绘、自动修改或作为主要视觉/文本来源的部分比例，而非仅作为参考的内容。（Note: The term “content proportion” here refers to the percentage of the final work that was directly generated, redrawn, or automatically modified by AI, or served as the primary source of visual or textual content—not content used merely as a reference.）

四、你的创作过程体验（Creative Process Experience）

（以下陈述请根据你的实际体验选择最符合的一项）
（Please select the statement that best matches your actual experience from the options below）

如果你未使用 AI，请根据你在创作过程中使用的**其他辅助工具或方法**作答；若某一陈述与你的情况明显不符，可选择“3 = 一般”。
（*If you did not use AI, please answer based on the **other tools or methods** you used during the creative process; if a statement clearly does not apply to your situation, you may select “3 = Neither agree nor disagree.”*）

说明（instructions）：
1 = 非常不同意　2 = 不同意　3 = 一般　4 = 同意　5 = 非常同意
（*1 = Strongly disagree　2 = Disagree　3 = Neither agree nor disagree　4 = Agree　5 = Strongly agree*）

（一）创作过程中的支持体验（Process Support)

1. AI 帮助我把复杂的创作任务拆分成更清晰的步骤
（AI helps me break down complex creative tasks into clearer steps）

1 ☐ 2 ☐ 3 ☐ 4 ☐ 5 ☐

2. 在不同创作阶段，我更清楚自己应该关注什么
（At different stages of the creative process, I have a clearer understanding of what I should focus on）

1 ☐ 2 ☐ 3 ☐ 4 ☐ 5 ☐

3. 使用 AI 后，我在操作或尝试上的心理负担减少了
（After using AI, I feel less psychological pressure when performing tasks or trying new approaches）

1 ☐ 2 ☐ 3 ☐ 4 ☐ 5 ☐

4. 当我遇到创作瓶颈时，AI 帮助我更快地继续推进
（When I hit a creative block, AI helps me move forward more quickly）

1 ☐ 2 ☐ 3 ☐ 4 ☐ 5 ☐

5. 我把 AI 当作一种支持工具，而不是代替我思考的工具
（I view AI as a support tool, not a tool that thinks for me）

1 ☐ 2 ☐ 3 ☐ 4 ☐ 5 ☐

（二）创意生成体验（Creative Process Experience）

1. 在本次创作中，我产生了比平时更多的想法或方案
（During this creative process, I generated more ideas or concepts than usual）

1 ☐ 2 ☐ 3 ☐ 4 ☐ 5 ☐

2. 我更容易尝试一些以前不会想到的创意方向
（I found it easier to explore creative directions I wouldn’t have considered before）

1 ☐ 2 ☐ 3 ☐ 4 ☐ 5 ☐

3. 我能更快地把抽象想法变成可见或可实现的形式
（I was able to turn abstract ideas into visible or actionable forms more quickly）

1 ☐ 2 ☐ 3 ☐ 4 ☐ 5 ☐

4. 我的创作探索范围变得更广，而不是很快固定在一个方案上
（My creative exploration became broader, rather than quickly settling on a single

solution）

1 ☐ 2 ☐ 3 ☐ 4 ☐ 5 ☐

（三）审美判断与选择（Aesthetic Judgment and Selection）

1. 我会主动判断不同版本在审美上是否符合我的目标
（I actively assess whether different versions meet my aesthetic goals）

1 ☐ 2 ☐ 3 ☐ 4 ☐ 5 ☐

2. 我能清楚说明为什么选择某一个版本而不是另一个
（I can clearly explain why I chose one version over another）

1 ☐ 2 ☐ 3 ☐ 4 ☐ 5 ☐

3. 我更注意作品在风格、构图或表达上的一致性
（I pay closer attention to consistency in style, composition, or expression within the work）

1 ☐ 2 ☐ 3 ☐ 4 ☐ 5 ☐

4. 我会根据审美判断主动调整作品，而不是直接接受结果
（I actively adjust the work based on my aesthetic judgment rather than simply accepting the result）

1 ☐ 2 ☐ 3 ☐ 4 ☐ 5 ☐

（四）技法学习与迁移（Learning and Transferring Techniques）

说明：“技法/方法”指可复用的创作流程、处理方式与决策策略（不只限于 AI 操作）。
（Note: “Techniques/methods” refer to reusable creative processes, approaches, and decision-making strategies (not limited to AI operations).）

1. 我在本次 AI 辅助创作中学到了一些可以在其他作品中继续使用的创作技法或方法
（Through this AI-assisted creative project, I have learned creative techniques or methods that I can continue to use in other works）

1 ☐ 2 ☐ 3 ☐ 4 ☐ 5 ☐

2. 即使不使用 AI，我也能复现或近似实现作品中的关键创作方法或处理方式
（Even without using AI, I can reproduce or approximate the key creative methods or

approaches used in this work）

1 ☐ 2 ☐ 3 ☐ 4 ☐ 5 ☐

3. 在本次创作中，我理解为什么要采用这些创作方法或处理方式，而不只是照着步骤完成
（In this creative process, I understood why these creative methods or approaches were chosen, rather than simply following the steps mechanically）

1 ☐ 2 ☐ 3 ☐ 4 ☐ 5 ☐

4. 我可以将这次学到的创作方法或处理方式迁移并应用到其他创作任务中
（I can transfer and apply the creative methods or techniques I learned in this project to other creative tasks）

1 ☐ 2 ☐ 3 ☐ 4 ☐ 5 ☐

（五）创作者身份与自主性（Authorship and Autonomy）

1. 我认为自己是这件作品的主要创作者
（I consider myself the primary creator of this work）

   1 ☐ 2 ☐ 3 ☐ 4 ☐ 5 ☐

2. 作品中重要的艺术决策是由我自己做出的
（I made the key artistic decisions for this work）

   1 ☐ 2 ☐ 3 ☐ 4 ☐ 5 ☐

3. 这件作品体现了我的个人表达和创作意图
（This work reflects my personal expression and creative intent）

   1 ☐ 2 ☐ 3 ☐ 4 ☐ 5 ☐

4. 使用 AI 并没有削弱我对自己创作能力的信心
（Using AI has not diminished my confidence in my own creative abilities）

   1 ☐ 2 ☐ 3 ☐ 4 ☐ 5 ☐

5. 我对最终完成的作品有明显的“拥有感”
（I have a strong sense of ownership over the final piece）

   1 ☐ 2 ☐ 3 ☐ 4 ☐ 5 ☐

6. 我能自主决定什么时候使用 AI、什么时候不用
（I can decide independently when to use AI and when not to）

   1 ☐ 2 ☐ 3 ☐ 4 ☐ 5 ☐

7. 当 AI 的结果不符合我的想法时，我会拒绝或修改
（When AI results don't align with my vision, I reject or modify them）

1 ☐ 2 ☐ 3 ☐ 4 ☐ 5 ☐

8. 在使用 AI 的过程中，我有时会感到自己更多是在“选择结果”，而不是在真正进行创作。
（While using AI, I sometimes feel like I'm mostly “selecting results” rather than truly creating. ）

1 ☐ 2 ☐ 3 ☐ 4 ☐ 5 ☐

9. 使用 AI 有时会让我怀疑这件作品是否还能完全代表“我自己”
（Using AI sometimes makes me wonder if the work can still fully represent “myself”）

1 ☐ 2 ☐ 3 ☐ 4 ☐ 5 ☐

五、学习收获与反思
（Learning Outcomes and Reflections）

1. 本次作业让我在艺术创作能力上有实际提升
（This assignment has helped me improve my artistic skills）

1 ☐ 2 ☐ 3 ☐ 4 ☐ 5 ☐

2. 我对今后独立完成类似创作更有信心
（I feel more confident about independently completing similar creative projects in the future）

1 ☐ 2 ☐ 3 ☐ 4 ☐ 5 ☐

3. 我对自己的创作过程有了更清晰的认识
（I have gained a clearer understanding of my creative process）

1 ☐ 2 ☐ 3 ☐ 4 ☐ 5 ☐

开放问题（简答）

1. 请描述你在本次创作中做出的一个**最重要的创作决策**，以及你为什么这样选择。（Please describe the most important creative decision you made during this project and explain why you made that choice.）

2. 如果你使用了 AI，你认为它在本次创作中**最有帮助的一点**是什么？它是否也存在一些局限？（If you used AI, what do you think was the most helpful aspect of it in this project? Were there any limitations?）
3. 你从这次作业中学到的最重要的一点是什么？是否可以应用到未来的创作中？（What is the most important thing you learned from this assignment? Can it be applied to future creative projects?）

六、对 AI 辅助创作后作品质量与展示意愿的看法 (Views on the Quality of AI-Assisted Work and Willingness to Share It)

以下问题主要了解你在 AI **辅助完成本次创作后**，
对自己作品质量的信心，以及你是否愿意将作品展示给更广泛的观众。
你的回答不会影响成绩，仅用于教学与研究分析。
（The following questions are designed to understand your confidence in the quality of your work after using AI to assist with this project, as well as your willingness to share it with a wider audience.Your answers will not affect your grade; they are used solely for teaching and research analysis.）

说明（Instructions）：
1 = 非常不同意　2 = 不同意　3 = 一般　4 = 同意　5 = 非常同意
（*1 = Strongly disagree　2 = Disagree　3 = Neither agree nor disagree　4 = Agree　5 = Strongly agree*）

（一）对作品质量的信心与判断

1. 我对本次完成的作品整体质量是有信心的
（I am confident in the overall quality of the work I have completed）

   1 ☐ 2 ☐ 3 ☐ 4 ☐ 5 ☐

2. 即使知道作品在某些阶段使用了 AI，我仍然认可其艺术完成度
（Even knowing that AI was used at certain stages, I still recognize the artistic merit of the work）

   1 ☐ 2 ☐ 3 ☐ 4 ☐ 5 ☐

3. 与以往不使用或较少使用 AI 的作品相比，我认为这次作品在质量上并不逊色
（Compared to previous works that did not use or used AI sparingly, I believe

this work is of comparable quality）

1 □ 2 □ 3 □ 4 □ 5 □

4. 我认为这件作品能够代表我当前的创作水平
（I believe this work represents my current level of artistic ability）

1 □ 2 □ 3 □ 4 □ 5 □

5. 如果不考虑课程要求，我也愿意将这件作品作为自己的正式创作成果之一
（Even without considering course requirements, I would be willing to include this work as one of my official creative outputs）

1 □ 2 □ 3 □ 4 □ 5 □

（二）对作品公开展示（线上 / 线下）的意愿（Willingness to publicly display the work (online/offline)）

6. 我愿意将本次作品用于课程相关的**线上展示**（如微信公众号、微博等）
（I am willing to have this work used for course-related online displays (e.g., WeChat Official Accounts, Weibo, etc.)）

1 □ 2 □ 3 □ 4 □ 5 □

7. 如果条件允许，我也愿意将本次作品用于**线下展览或公开展示**
（If conditions permit, I am also willing to use this work for offline exhibitions or public displays）

1 □ 2 □ 3 □ 4 □ 5 □

8. 我对他人（同学、教师或公众）看到这件作品持较为积极的态度
（I have a generally positive attitude toward others (classmates, teachers, or the public) seeing this work）

1 □ 2 □ 3 □ 4 □ 5 □

9. 即使他人知道作品在创作过程中使用了 AI，我仍愿意公开展示它
（I am still willing to publicly display this work even if others know that AI was used in its creation）

1 □ 2 □ 3 □ 4 □ 5 □

10. 我认为 AI 辅助并不会降低作品在公开展示中的专业性或严肃性
（I do not believe that AI assistance diminishes the professionalism or seriousness of the work when it is publicly displayed）

1 □ 2 □ 3 □ 4 □ 5 □

11. 我不愿公开展示作品主要是因为隐私、版权或平台规则等现实顾虑（不一定是对作品质量缺乏信心）。
（My reluctance to publicly display my work stems primarily from practical concerns such as privacy, copyright, or platform rules (not necessarily a lack of confidence in the quality of the work).）

1 ☐ 2 ☐ 3 ☐ 4 ☐ 5 ☐

开放问题（Open-ended Questions）

12. 如果你的作品被选用于线上或线下展览，你希望观众**如何理解或关注这件作品**？（例如：创意、风格、主题、技术、情感表达等）
（If your work is selected for an online or in-person exhibition, how would you like the audience to interpret or engage with it?(e.g., creativity, style, theme, technique, emotional expression, etc.)）
13. 你是否对“AI 辅助创作的作品被公开展示”有任何顾虑或期待？请简单说明。
（Do you have any concerns or expectations regarding the public display of AI-assisted works? Please briefly explain.）

七、对创作选题延展与社会应用的意愿与看法（Willingness and Views on Expanding the Creative Theme and Its Social Applications）

以下问题主要了解你在完成本次 AI 辅助艺术创作后，
是否有意愿在该选题基础上继续深化创作，以及你对作品**社会应用与文化传播价值**的看法。
本部分仅了解你对创作延展与社会应用的个人想法，不涉及作品是否“应当”服务社会，也不影响课程评价。
（The following questions are designed to understand whether, after completing this AI-assisted art creation project, you are willing to further develop your work based on this theme, as well as your views on the social applications and cultural dissemination value of the work.This section is intended solely to gather your personal thoughts on expanding the creative work and its social applications; it does not address whether the work “should” serve society, nor does it affect your course evaluation.）

说明（Instructions）：
1 = 非常不同意　2 = 不同意　3 = 一般　4 = 同意　5 = 非常同意

（*1 = Strongly disagree　2 = Disagree　3 = Neither agree nor disagree　4 = Agree　5 = Strongly agree*）

（一）对选题持续深化的意愿（创作纵向延展）（Willingness to continue exploring the topic in depth (longitudinal expansion of the creative project）

1. 完成本次作业后，我仍然对这一创作选题保持兴趣
（After completing this assignment, I remain interested in this creative topic）

1 ☐ 2 ☐ 3 ☐ 4 ☐ 5 ☐

2. 我愿意在本次作品的基础上继续深化这一创作方向
（I am willing to build upon this work to further explore this creative direction）

1 ☐ 2 ☐ 3 ☐ 4 ☐ 5 ☐

3. 如果课程或项目允许，我希望将该作品发展为系列作品或长期项目
（If the course or project allows, I hope to develop this work into a series or long-term project）

1 ☐ 2 ☐ 3 ☐ 4 ☐ 5 ☐

4. 我认为这一选题具有进一步探索的空间，而不仅适合作为一次性作业
（I believe this topic has room for further exploration and is not merely suitable as a one-time assignment）

1 ☐ 2 ☐ 3 ☐ 4 ☐ 5 ☐

（二）对现实机构对接与合作的意愿（Willingness to Collaborate with Actual Institutions）

本部分所指的机构包括：**博物馆、美术馆、文化馆、文化旅游部门、广播电视与新媒体机构等**。
（The institutions referred to in this section include: museums, art galleries, cultural centers, cultural tourism departments, radio and television stations, and new media organizations.）

5. 我愿意将本次作品或相关创作方向与实际文化机构进行对接
（I am willing to collaborate with actual cultural institutions regarding this work or related creative directions）

1 ☐ 2 ☐ 3 ☐ 4 ☐ 5 ☐

6. 如果有机会，我愿意根据真实机构需求对作品进行修改或再创作
（If given the opportunity, I am willing to modify or rework the work based on the actual needs of the institution）

1 ☐ 2 ☐ 3 ☐ 4 ☐ 5 ☐

7. 我对自己的作品被用于公共文化传播持开放态度
（I am open to my work being used for public cultural dissemination）

1 ☐ 2 ☐ 3 ☐ 4 ☐ 5 ☐

8. 我认为本次作品具备与现实文化项目对接的潜力
（I believe this work has the potential to align with real-world cultural initiatives）

1 ☐ 2 ☐ 3 ☐ 4 ☐ 5 ☐

（三）对作品社会实效性与功能属性的看法

（Views on the Social Impact and Functional Attributes of Artworks）

（侧重作品在文化传播与转化中的作用）（Focusing on the role of artworks in cultural dissemination and transformation）

9. 我在创作过程中考虑过作品是否有助于文化传播或文化表达
（During the creative process, I considered whether the artwork contributes to cultural dissemination or cultural expression）

1 ☐ 2 ☐ 3 ☐ 4 ☐ 5 ☐

10. 我认为艺术作品不仅具有审美价值，也可以承担一定的社会或文化功能
（I believe that works of art not only possess aesthetic value but can also fulfill certain social or cultural functions）

1 ☐ 2 ☐ 3 ☐ 4 ☐ 5 ☐

11. 在本次创作中，我有意识地思考作品是否能够被不同受众理解或接受
（In this creative project, I consciously considered whether the work could be understood or accepted by different audiences）

1 ☐ 2 ☐ 3 ☐ 4 ☐ 5 ☐

12. 我认为 AI 辅助创作有可能帮助艺术作品更好地服务文化传播或文化转化
（I believe AI-assisted creation has the potential to help artworks better serve cultural dissemination or cultural transformation.）

1 ☐ 2 ☐ 3 ☐ 4 ☐ 5 ☐

13. 如果作品被用于文化展示、传播或公共服务场景，我愿意参与相关调整或解释工作

（If the work is used in cultural exhibitions, dissemination, or public service contexts, I am willing to participate in related adjustments or explanatory work.）

1 ☐ 2 ☐ 3 ☐ 4 ☐ 5 ☐

（四）创作后的进一步设想 （Future Directions for the Project ）

14. 如果你继续深化这一创作选题，你最希望它向哪个方向发展？（如主题深化、媒介扩展、受众拓展、社会应用等）
（If you were to further develop this project, in which direction would you most like to see it evolve?(e.g., deepening the theme, expanding the medium, broadening the audience, or exploring social applications)）
15. 你认为本次作品在文化传播、文化教育或文化转化方面，可能具有什么样的实际价值？
（What practical value do you believe this work might hold in terms of cultural dissemination, cultural education, or cultural transformation?）

八、对本次创作作业与课程教学方式的评价（Evaluation of This Creative Assignment and Teaching Methods）

以下问题主要了解你对本次期末艺术创作作业以及**教师鼓励使用 AI 进行创作**这一教学方式的看法，请根据你的**真实体验作答**，而不是基于你认为课程或教师所期待的回答。
请你将以下问题视为**对课程体验的独立评价**，而不是对你个人创作表现的评价。
你的回答不会影响成绩，仅用于改进课程设计与教学研究。
（The following questions are designed to understand your views on this final art creation assignment and the teaching approach that encourages the use of AI in the creative process. Please answer based on your actual experience, rather than on what you think the course or instructor expects. Please treat the following questions as an independent evaluation of your course experience, not as an assessment of your personal creative performance.Your responses will not affect your grade; they are solely for the purpose of improving course design and teaching research.）

说明 （Instructions）：
1 = 非常不同意　2 = 不同意　3 = 一般　4 = 同意　5 = 非常同意

（*1 = Strongly disagree　2 = Disagree　3 = Neither agree nor disagree　4 = Agree　5 = Strongly agree*）

（一）对本次艺术创作作业的自我评价（Self-Evaluation of This Art Project）

1. 我认为自己在本次期末创作作业中投入了充分的时间和精力
（I believe I devoted sufficient time and effort to this final project）

   1 ☐ 2 ☐ 3 ☐ 4 ☐ 5 ☐

2. 本次作业较好地体现了我的真实创作水平
（This assignment accurately reflects my true creative ability）

   1 ☐ 2 ☐ 3 ☐ 4 ☐ 5 ☐

3. 与课程开始时相比，我认为自己的创作能力有所提升
（Compared to the beginning of the course, I believe my creative abilities have improved）

   1 ☐ 2 ☐ 3 ☐ 4 ☐ 5 ☐

4. 本次作业让我对艺术创作过程有了更深入的理解
（This assignment has given me a deeper understanding of the artistic creation process）

   1 ☐ 2 ☐ 3 ☐ 4 ☐ 5 ☐

5. 本次作业让我更清楚自己的优势与不足
（This assignment has helped me better understand my strengths and weaknesses）

   1 ☐ 2 ☐ 3 ☐ 4 ☐ 5 ☐

6. 整体而言，我对自己本次提交的作品是满意的
（Overall, I am satisfied with the work I submitted）

   1 ☐ 2 ☐ 3 ☐ 4 ☐ 5 ☐

（二）对教师鼓励使用 AI 进行创作的教学方式的评价（**Evaluation of Teaching Methods That Encourage the Use of AI for Creative Work**）

1. 教师鼓励使用 AI 进行创作让我感到学习环境更加开放
（The teacher’s encouragement to use AI for creative work makes me feel

that the learning environment is more open）

1 ☐ 2 ☐ 3 ☐ 4 ☐ 5 ☐

2. 在本课程中，我感到是否使用 AI 是由我自主决定的
（In this course, I feel that the decision to use AI is entirely up to me）

1 ☐ 2 ☐ 3 ☐ 4 ☐ 5 ☐

3. 教师对 AI 的态度让我更愿意主动探索新的创作方式
（The teacher’s attitude toward AI makes me more willing to actively explore new creative approaches）

1 ☐ 2 ☐ 3 ☐ 4 ☐ 5 ☐

4. 教师鼓励使用 AI 有助于我更专注于创意和表达本身
（The instructor’s encouragement to use AI helped me focus more on creativity and expression itself）

1 ☐ 2 ☐ 3 ☐ 4 ☐ 5 ☐

5. 在课程中使用 AI 并没有让我感到被“要求依赖”或被强迫使用
（Using AI in this course did not make me feel “required to rely on” or forced to use it）

1 ☐ 2 ☐ 3 ☐ 4 ☐ 5 ☐

6. 我认为教师对 AI 的引导方式有助于负责任地使用技术
（I believe the way teachers guide students in using AI promotes responsible technology use）

1 ☐ 2 ☐ 3 ☐ 4 ☐ 5 ☐

7. 总体而言，我认可在艺术创作课程中鼓励但不强制使用 AI 的教学方式
（Overall, I approve of the teaching approach that encourages but does not mandate the use of AI in art creation courses）

1 ☐ 2 ☐ 3 ☐ 4 ☐ 5 ☐

简答题（Short Answer Questions）

- 你对本次期末艺术创作作业还有哪些意见或建议？（如作业形式、难度、时间安排等）
（Do you have any other comments or suggestions regarding this final art creation assignment?(e.g., assignment format, difficulty, schedule, etc.)）
- 你对教师在课程中引导和鼓励使用 AI 进行创作，有哪些看法或建议？
（What are your thoughts or suggestions regarding teachers guiding and encouraging the use of AI for creative work in the course?）
